# Preferential Genome Targeting of the CBP Co-Activator by Rel and Smad Proteins in Early *Drosophila melanogaster* Embryos

**DOI:** 10.1371/journal.pgen.1002769

**Published:** 2012-06-21

**Authors:** Per-Henrik Holmqvist, Ann Boija, Philge Philip, Filip Crona, Per Stenberg, Mattias Mannervik

**Affiliations:** 1 The Wenner-Gren Institute, Developmental Biology, Stockholm University, Stockholm, Sweden; 2 Department of Molecular Biology, Umeå University, Umeå, Sweden; 3 Computational Life Science Cluster (CLiC), Umeå University, Umeå, Sweden; Harvard Medical School, Howard Hughes Medical Institute, United States of America

## Abstract

CBP and the related p300 protein are widely used transcriptional co-activators in metazoans that interact with multiple transcription factors. Whether CBP/p300 occupies the genome equally with all factors or preferentially binds together with some factors is not known. We therefore compared *Drosophila melanogaster* CBP (*nejire*) ChIP–seq peaks with regions bound by 40 different transcription factors in early embryos, and we found high co-occupancy with the Rel-family protein Dorsal. Dorsal is required for CBP occupancy in the embryo, but only at regions where few other factors are present. CBP peaks in mutant embryos lacking nuclear Dorsal are best correlated with TGF-ß/Dpp-signaling and Smad-protein binding. Differences in CBP occupancy in mutant embryos reflect gene expression changes genome-wide, but CBP also occupies some non-expressed genes. The presence of CBP at silent genes does not result in histone acetylation. We find that Polycomb-repressed H3K27me3 chromatin does not preclude CBP binding, but restricts histone acetylation at CBP-bound genomic sites. We conclude that CBP occupancy in *Drosophila* embryos preferentially overlaps factors controlling dorso-ventral patterning and that CBP binds silent genes without causing histone hyperacetylation.

## Author Summary

Development of an embryo into different cell types relies on regulation of gene expression, whereby genes are coordinately turned on or off. CBP and the related p300 protein are central regulators of gene expression in animal cells. These co-activator proteins facilitate gene activation by a multitude of transcription factors, possibly through their histone acetyltransferase activity. Consequently, loss of CBP/p300 gene function disrupts development and is lethal in mice, worms, and flies. How CBP/p300 is targeted to regulatory DNA sequences is not understood. Here, we have compared genome occupancy of CBP with 40 different transcription factors in *Drosophila* embryos and find that master regulators of dorsal-ventral patterning, the transcription factors Dorsal and Medea, target CBP to the genome. CBP occupies mainly active genes in embryos, where histones become acetylated. Surprisingly, the presence of CBP at silent genes does not result in histone acetylation. We find that repressive chromatin prevents histone acetylation by CBP. Our results demonstrate that CBP preferentially associates with some gene regulatory networks and that CBP binds silent genes without causing histone acetylation. These data have implications for prediction of transcriptional regulatory sequences and for understanding gene regulation by one of the most widely used co-activators in animal cells.

## Introduction

CREB-binding protein (CBP) and its paralog p300 are widely used transcriptional co-regulators with histone acetyltransferase (HAT) activity (reviewed in [1]). Over 400 interaction partners have been described for these proteins, including transcription factors of all major families, and they are therefore believed to be present at many transcriptional regulatory regions. Indeed, chromatin immunoprecipitation (ChIP) of p300/CBP has been used to successfully predict novel enhancers (e.g. [2], [3]). Although p300/CBP can interact with most transcription factors *in vitro*, it is not known whether p300/CBP preferentially associates with some factors *in vivo*. Here, we use the early *Drosophila melanogaster* embryo to compare the genomic distribution of p300/CBP with 40 transcription factors involved in embryonic patterning and cell differentiation.

*Drosophila* has one CBP/p300 ortholog, also known as nejire [4]. Chromatin binding of *Drosophila* CBP has recently been used to identify novel enhancers that are active in embryos [5]. By comparing CBP occupancy at different stages of *Drosophila* development, around 14 000 CBP peaks were identified that may represent regulatory DNA sequences. CBP binding was found to correlate with active chromatin, including histone acetylation and H3K4 methylation [5]. *Drosophila* CBP has been implicated in Hedgheog, Wnt, and TGF-ß signaling, as well as in dorsal-ventral patterning of early embryos [reviewed in 6]. The loss of function allele *nejire^3^* (*nej^3^*) is cell-lethal, whereas the hypomorphic *nej^1^* allele reduces CBP expression approximately two-fold, and causes embryonic patterning phenotypes [7]–[11]. These can be attributed to reduced signaling by the TGF-ß molecule Decapentaplegic (Dpp), in turn caused by impaired expression of the Tolloid (Tld) protease in *nej^1^* embryos [10]. In the absence of Tld, the Short-gastrulation (Sog) inhibitor prevents the Dpp ligand from signaling through its receptors. Interestingly, the acetyltranferase activity of CBP appears dispensable for *tld* gene activation [9].

Embryonic dorsal-ventral patterning is controlled by an intra-nuclear concentration gradient of Dorsal, a Rel-family transcription factor related to NF-κB. Over 50 Dorsal target genes are known, constituting one of the best understood gene regulatory networks in animal development (reviewed by [12]). Dorsal enters ventral nuclei at high levels in response to signaling by the transmembrane receptor Toll. The Toll ligand Spätzle is present in the periviteline space surrounding the embryo, at high concentrations on the ventral side and progressively lower concentration in lateral and dorsal regions [13]. A proteolytic cascade is responsible for generating active Spätzle ligand, and mutations that disrupt this cascade, such as in the Pipe sulfotransferase and in the protease Gastrulation defective (gd), result in absence of Toll signaling and failure of Dorsal to enter the nucleus (reviewed in [14]). In such mutants, the entire embryo is converted to presumptive dorsal ectoderm tissue. By contrast, a constitutively active form of Toll [15], *Toll^10B^*, results in high Dorsal concentration in all embryonic nuclei, generating embryos consisting entirely of presumptive mesoderm. In embryos derived from *Toll^rm9/rm10^* mutant mothers [15], Dorsal enters all nuclei at an intermediate level corresponding to that found in the lateral, neuroectoderm region. Dorsal regulates gene expression in a concentration-dependent manner (reviewed by [16]). Target genes such as *twist (twi)* and *snail (sna)* with low-affinity bindning sites are turned on in ventral, presumptive mesodermal cells where Dorsal concentration is highest. When Dorsal sites are positioned in proximity to AT-rich binding sites, Dorsal is converted to a repressor that recruits the co-repressor Groucho and thereby prevents expression of dorsal ectoderm targets such as *zerknüllt* (*zen*), *tld*, and *dpp* in lateral and ventral parts of the embryo [17]–[19]. This restricts Dpp-signaling to the dorsal ectoderm where it sub-divides this tissue by regulating gene expression in a concentration-dependent manner [8].

CBP can function as a Dorsal co-activator as they genetically interact and bind each other *in vitro* [7]. However, to what extent Dorsal relies on CBP for gene activation *in vivo* is not known. Here, we describe a high concordance in genome occupancy of CBP and Dorsal. We show that CBP occupancy differs in mutant embryos where Dorsal fails to enter the nucleus, and that this difference often correlates with changes in gene expression. Moreover, CBP occupancy in mutant embryos coincides with regions bound by the Smad protein Medea, a mediator of Dpp-signaling (reviewed in [20]). Thus, genome occupancy of CBP is most strongly associated with dorsal-ventral axis specification in *Drosophila* embryos, consistent with earlier studies on CBP mutant phenotypes. Although CBP associates with some Dorsal-target genes in tissues where they are not expressed, this does not result in histone acetylation. We find that Polycomb-repressed H3K27me3 chromatin is present at the Dorsal-target genes, which does not preclude CBP binding, but restricts histone acetylation at these CBP-bound genomic sites.

## Results

### CBP-binding sites highly overlap Dorsal-bound genomic regions in early *Drosophila* embryos

We compared the published CBP binding data in *Drosophila* 0–4 hour embryos [5] to regions bound by 40 sequence-specific transcription factors mapped at a similar stage of embryo development [21], [22]. There is a particularly strong correlation between genome occupancy of CBP and the key activator of dorsal-ventral patterning, the transcription factor Dorsal. Eighty-two percent of the CBP peaks overlap a Dorsal-bound region (Table S1). To normalize for different number of identified regions for the 40 factors, we used the 300 most strongly bound regions for each factor in comparison with all CBP peaks. This shows that the CBP peaks still overlap best with regions occupied by Dorsal (Table S1). To further investigate this overlap of CBP and Dorsal occupancy, we performed CBP ChIP-seq with chromatin extracts from 2–4 hour old wild-type and mutant embryos where Dorsal fails to enter the nucleus (*gd^7^*). The CBP serum is affinity-purified and CBP-specific, and quantitatively similar levels of CBP occupancy is found at several loci with another CBP antibody (Figure S1). To calculate peaks and bound regions, the 5% highest enrichment values in both wild-type and *gd^7^* embryos were extracted, corresponding to a cut-off of 1.9 in wild-type and 1.9 in *gd^7^*. High-confidence peaks were then defined as regions of at least 200 bp with enrichment values of at least 1.9 (in log_2_ scale).

**Figure 1:**
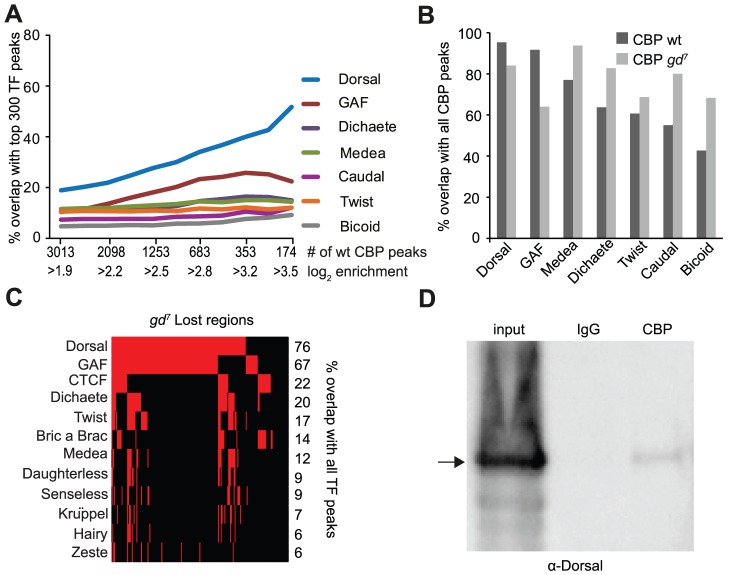
CBP and Dorsal co-occupy the genome more extensively than 39 other transcription factors in early *Drosophila* embryos. A) Percent overlap of CBP peaks in *Drosophila* 2–4 hour embryos with the 300 regions occupied most strongly by transcription factors at a similar stage of development. The peaks were selected with increasing cut-off, so that all CBP peaks are used for the comparison in the leftmost value on the x-axis, and increasing cut-offs leading to fewer, but stronger peaks along the x-axis. Note that long binding regions for one factor can overlap several CBP peaks. Only seven out of 40 transcription factors are shown. The CBP peaks overlap Dorsal bound regions more than any other factor. B) Percent overlap of the 300 strongest regions for seven transcription factors with all 3013 CBP peaks in wild-type and 1939 peaks in mutant embryos where Dorsal cannot enter the nucleus (*gd^7^*). Dorsal-binding regions overlap the CBP peaks better than any other factor in wild-type. C) Genomic regions where CBP occupancy is lost in *gd^7^* mutant embryos (n = 191) were compared to all peaks for 40 transcription factors in wild-type embryos. Presence of a factor is indicated by red and absence by black. Only factors with >5% overlap are shown. Dorsal binds to 76% of the regions where CBP occupancy is lost in *gd^7^* embryos. D) Dorsal can be co-immunoprecipitated with CBP from wild-type embryos. The CBP antibody and control immunoglobulin G (IgG) were used in immunoprecipitation from 2–4 hour old embryo extracts, separated on a gel together with 10% input, and probed with a Dorsal antibody. A fraction of Dorsal is associated with CBP.

We identified 3013 high-confidence peaks in wild-type and 1939 CBP peaks in *gd^7^* embryos. These CBP peaks were compared to the occupancy of the previously mapped 40 transcription factors [21], [22]. We divided the CBP peaks into bins of increasing cut-off, so that fewer but stronger CBP peaks are shown along the x-axis, and plotted the overlap with the 300 strongest regions for each factor. As shown in Figure 1A and Table S2, the CBP-peaks in wild-type embryos overlap most extensively with Dorsal. Furthermore, the stronger the CBP-peaks, the better the overlap with Dorsal. Fifty-two percent of the 174 strongest CBP-peaks overlap the top 300 Dorsal-bound regions (Figure 1A). By contrast, the CBP peaks do not overlap regions bound by transcriptional activators that pattern the anterior-posterior axis, such as Bicoid and Caudal, to more than 10–15 percent (Figure 1A). Similar results were obtained with unprocessed CBP data, showing that the high overlap with Dorsal is not due to the way we defined the CBP-bound regions (Table S3). We then determined how many of the 300 strongest regions for each factor overlap all CBP peaks in wild-type. As shown in Figure 1B, 95% of the 300 strongest Dorsal peaks overlap any of the 3013 regions bound by CBP in wild-type embryos. Thus, virtually all of the strong Dorsal-binding regions in the genome are also occupied by CBP. Since a lot of transcription factors bind to many of the same genomic sites [reviewed in 23], we expected other factors to overlap the CBP peaks to a similar degree as Dorsal. However, we find that other factors do not overlap CBP-bound regions to the same extent as Dorsal in wild-type embryos, although GAGA-factor (GAF) binding regions also overlap the CBP-peaks extensively (Figure 1A, 1B and Table S4). We conclude that Dorsal and GAF are associated with CBP to a larger extent than other factors in early embryos.

To investigate if Dorsal is required for CBP's association with the genome, we next compared CBP occupancy in wild-type and *gd^7^* mutant embryos. As shown in Figure 1B, fewer of the Dorsal and GAF binding regions overlap CBP peaks in *gd^7^* mutant embryos than CBP peaks in wild-type. By contrast, many other factors overlap the *gd^7^* CBP peaks better than wild-type peaks. This indicates that CBP occupies regions bound by multiple factors to a larger extent in *gd^7^* than in wild-type embryos (see below). In *gd^7^* embryos that lack Dorsal in the nucleus, CBP continues to associate with 84% of Dorsal-binding regions, suggesting that other factors maintain CBP binding at these places in the absence of Dorsal. However, CBP occupancy at the top 300 Dorsal-binding regions is significantly lower in *gd^7^* embryos compared to wild-type (paired T-test *p* = 5.22×10^−9^), showing that CBP occupies many Dorsal targets in *gd^7^* embryos less strongly. We therefore extracted the regions where the CBP-peaks were completely lost in *gd^7^* embryos, and compared them to all of the regions bound by the 40 factors in wild-type (not only the top 300 regions). High-confidence CBP peaks in wild-type with a CBP enrichment of less than 0.5 (in log_2_ scale) in *gd^7^* were considered to be completely lost. It was found that 76% and 67% of the peaks that are lost in *gd^7^* embryos overlap Dorsal or GAF respectively, but that no other factor overlaps these CBP-peaks to more than 22% (Figure 1C and Table S5). This indicates that Dorsal is important for targeting CBP to chromatin in *Drosophila* embryos, and since Dorsal and GAF co-occupy many of these regions (Figure 1C), that GAF may cooperate with Dorsal in specifying CBP binding. CBP and Dorsal can be co-immunoprecipitated from wild-type embryos (Figure 1D), indicating that Dorsal may directly bring CBP to its genomic binding sites. Taken together, our results suggest that Dorsal targets CBP to many sites throughout the genome.

### Dpp-target genes are highly occupied by CBP in embryos lacking nuclear Dorsal

**Figure 2:**
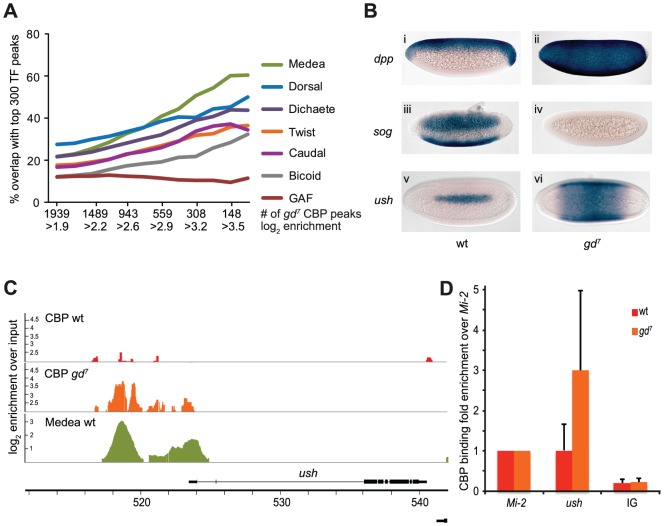
CBP binds to Dpp-target genes in *gd^7^* mutant embryos. A) Percent overlap of CBP peaks in 2–4 hour *gd^7^* mutant embryos with the 300 strongest binding regions of transcription factors in wild-type embryos. Seven out of 40 factors are shown. The stronger CBP peaks overlap regions bound by the Smad4 protein Medea, a transducer of Dpp-signaling, more than any other factor. B) Dorsal fails to enter the nucleus in *gd^7^* mutant embryos, resulting in conversion of the entire embryo into dorsal ectoderm tissue where signaling by the TGF-ß molecule Dpp occurs. Consequently, expression of *dpp* is expanded (ii), expression of the Dpp-inhibitor *sog* absent (iv), and expression of the Dpp-target gene *ush* expanded (vi) in *gd^7^* mutant embryos compared to wild-type (i, iii, v). Two to four hour old embryos were hybridized with digoxigenin-labeled probes and are oriented with anterior to the left. Lateral views with dorsal up are shown in (i) and (ii), ventro-lateral views in (iii) and (iv), and dorsal views are shown in (v) and (vi). C) CBP and Medea occupancy overlap at the *ush* locus, a Dpp-target gene. CBP ChIP-seq peaks (as defined in Materials &Methods) in wild-type (wt) and *gd^7^* mutant embryos, as well as Medea ChIP-chip peaks in wt are shown. Occupancy is plotted as log_2_-fold enrichment over input. D) ChIP-qPCR confirms that CBP occupancy is elevated at the *ush* locus in *gd^7^* embryos (T-test *p* = 0.045). CBP occupancy is plotted as fold enrichment relative the *Mi-2* locus. Background occupancy levels are detected at two intergenic loci, the average of which is plotted (IG). Mean fold enrichment and standard deviations from 6 (wt) or 5 (*gd^7^*) independent biological replicates are shown.

To identify Dorsal-independent transcription factor sites that are co-occupied by CBP, we next compared the CBP peaks in *gd^7^* embryos to regions bound by the 40 transcription factors in wild-type embryos. Figure 2 shows that the best overlap of the strong CBP peaks in *gd^7^* embryos is with the Smad4 protein Medea (Med), a transducer of Dpp-signaling (Figure 2A and Table S2). In *gd^7^* embryos, all cells are converted to dorsal ectoderm, the tissue where Dpp-signaling occurs. Consequently, expression of *dpp* is expanded, whereas the expression of the Dpp-inhibitor *sog* is absent (Figure 2B). This results in expanded expression of Dpp-target genes in *gd^7^* mutant embryos (*u-shaped (ush)* in Figure 2B, as well as *Race* and *pannier* in Figure S2). In wild-type embryos, these Dpp-target genes are expressed in a restricted number of cells in dorsal parts of the embryo, whereas in *gd^7^* mutants they become expressed throughout the entire circumference of the embryo. Thus, Dpp-signaling occurs in the entire embryo in *gd^7^* mutants, thereby providing a genetic background in which binding of CBP to Dpp-target genes can be visualized.

As illustrated in Figure 2C, CBP occupancy of the Dpp-target gene *ush* is hardly detectable in wild-type embryos, but highly evident in *gd^7^* embryos. Similar results were obtained for other Dpp-target genes, including *Race*, *tail-up*, *GATAc*, and *pannier* (Figure S2). As expected, the CBP peaks are found at Med-binding regions at Dpp-target genes (Figure 2C and Figure S2). We confirmed that CBP occupies Dpp-target genes to a larger extent in *gd^7^* embryos than in wild-type by ChIP-qPCR (Figure 2D and Figure S2, T-test of CBP at *ush* in *gd^7^* vs wild-type, *p* = 0.045, at *pnr p* = 0.012). Our results indicate that CBP becomes recruited to Dpp-target genes upon signaling, consistent with previous observations that Dpp-signaling is impaired in CBP mutant embryos [8], [10], [11]. We conclude that whereas Dorsal and CBP binding strongly coincide genome-wide in wild-type embryos, CBP associates with Smad binding sites genome-wide upon increased TGF-ß signaling. This shows that the gene regulatory networks controlled by the two key morphogens in dorsal-ventral patterning, Toll/Dorsal and Dpp/Medea, are to a larger extent than others associated with CBP in early embryos.

### In embryos lacking nuclear Dorsal, CBP occupancy is reduced at regions where Dorsal, but few other factors, binds in wild-type embryos

**Figure 3:**
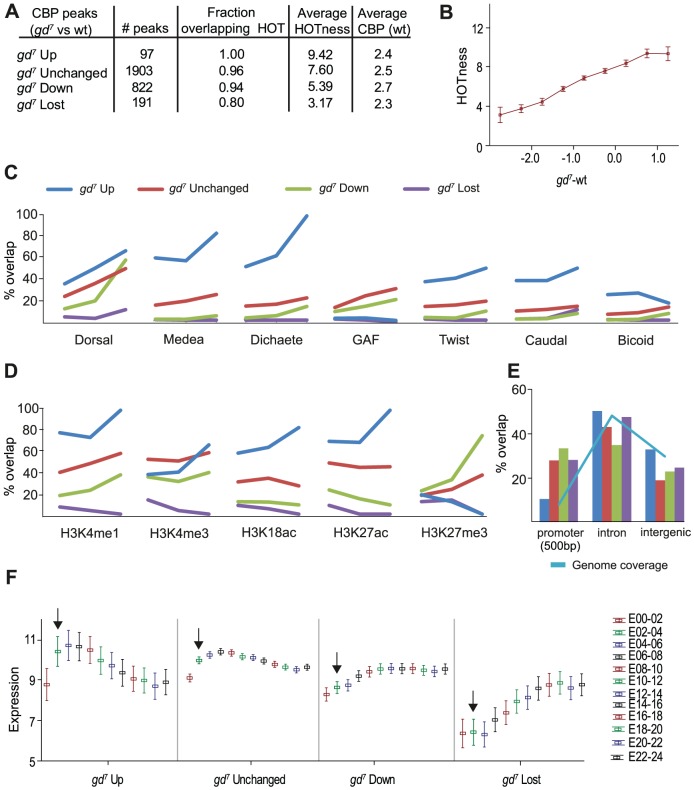
CBP peaks can be classified based on their change in *gd^7^* embryos. CBP peak values in *gd^7^* embryos were calculated within each CBP bound region from wild-type embryos. Up and down represent regions where CBP occupancy is altered more than 2-fold, unchanged are regions with less than a 2-fold change. In regions for which the *gd^7^* peak values were below 0.5 fold enrichment in log_2_ scale, CBP was considered lost. A) Number of peaks for each category, their overlap with High Occupancy Target (HOT) regions, mean HOTness, and mean fold enrichment over input of wild-type CBP peaks in log_2_ scale. B) Fold-change (log_2_) in CBP occupancy between *gd^7^* and wild-type plotted versus HOTness. Squares indicate mean HOTness and whiskers correspond to 95% confidence intervals. The less CBP occupancy in *gd^7^* compared to wild-type, the lower the HOTness of the region. C–E) *gd^7^* Up is shown in blue, Unchanged in red, Down in green, and Lost in purple. C) Percent overlap of the four categories of CBP peaks with the 300 strongest binding regions for each transcription factor in wild-type embryos. The three cut-offs for the CBP peaks are; 1) no cut-off (all regions included), 2) an average of cut-offs 1 and 3, 3) the highest cut-off corresponding to the ∼5% most enriched peaks in wild-type embryos for each category. D) Percent overlap of the four categories of CBP peaks with histone modifications in 0–4 hour embryos (H3K18ac in 2–4 hour embryos). Cut-offs as in C). E) Percent overlap of the four categories of CBP peaks with gene features. Promoters (+/−500 bp of transcription start site, TSS), introns, and intergenic regions are shown. Less than 3% of the peaks overlap coding regions and 3′UTRs and are therefore not shown. Overlap with 5′UTRs may result from CBP binding close to the TSS in promoters and is not included. The genome coverage illustrates how common each gene feature is in the genome and is shown in light blue. F) Mean expression of genes associated with the four categories of CBP peaks at different stages of embryo development. Each peak was associated with the closest TSS, and expression of the corresponding gene included in the analysis [46]. Log_2_ transformed expression values are plotted against developmental time point (0–2 hour embryos (E00-02), etc). Whiskers correspond to 95% confidence intervals. Arrows point to expression in 2–4 hour old embryos.

We next separated the CBP-peaks into those that remain unchanged between wild-type and *gd^7^*, those where CBP binding increases at least 2-fold in *gd^7^* versus wild-type (*gd^7^* Up), those where CBP binding decreases at least 2-fold in *gd^7^* versus wild-type (*gd^7^* Down), and those that are completely lost in *gd^7^*. We first looked at how many other factors that occupy these CBP-bound regions, measured in their HOTness. High occupancy target (HOT) regions are defined as genomic sites binding at least one of the 40 transcription factors [22]. The minimum HOTness value is 1 and increases with the number of factors and the number of sites for each factor found within the genomic region. Very HOT regions, or hotspots, are found across the *Drosophila*, *C. elegans*, and human genomes, and are associated with open chromatin, but their function is not understood [22]. As shown in Figure 3A, most CBP-peaks overlap a HOT region. CBP-peaks that increase in *gd^7^* have a high HOTness, whereas unchanged, down and lost in *gd^7^* are decreasingly HOT. This is also illustrated in Figure 3B, where the change in CBP occupancy in *gd^7^* versus wild-type is plotted against HOTness. Remarkably, there is an almost perfect correlation between the difference in CBP occupancy in *gd^7^* versus wild-type embryos and mean HOTness. Thus, in *gd^7^* embryos, CBP is present at regions where many factors are bound, but decreases or is lost at regions bound by only few factors. However, the highest mean CBP occupancy in wild-type is found at regions where CBP binding decreases in *gd^7^* embryos (Figure 3A), suggesting that HOTness alone does not determine how much CBP that binds a particular genomic region. Rather, the HOTness determines if CBP occupancy will change in the absence of Dorsal.

We then compared the different classes of CBP-bound regions with the top 300 regions bound by the 40 transcription factors, as well as with histone modifications, gene features, and gene expression. We divided the CBP peaks into three bins of increasing cut-off, so that fewer but stronger CBP peaks are shown along the x-axis. The first bin represents all CBP peaks within the respective class, and the third bin the ∼5% strongest peaks. For the second bin we used an enrichment cut-off midway between the cut-offs for bin one and three. The bins were used to calculate % overlap with other genomic features.

Regions where CBP binding increases in *gd^7^* mutants overlap most factors extensively, consistent with the *gd^7^* Up regions having the highest HOTness (Figure 3A and 3C). However, the *gd^7^* Up regions do not overlap the top 300 GAF-binding sites (Figure 3C). As expected from the analysis in Figure 2, CBP-peaks that increase in *gd^7^* show a high overlap with Medea. Surprisingly, the *gd^7^* Up peaks that are strongest in wild-type overlap the Sox-protein Dichaete even better than binding regions for Medea (Figure 3C). Dichaete is involved in both anterior-posterior patterning by regulating pair-rule gene expression, and in dorsal-ventral pattering where it is expressed in medial and lateral regions of the neuroectoderm [reviewed by 24]. Our results indicate that Dichaete-regulated genes become associated with CBP in *gd^7^* embryos.

CBP-peaks that increase in strength in *gd^7^* embryos (*gd^7^* Up) strongly overlap H3K4me1, a histone modification associated with enhancer sequences, as well as the “active” histone marks H3K18ac and H3K27ac, but are depleted of H3K27me3-repressed chromatin (Figure 3D). Almost all of the *gd^7^* Up CBP-peaks map to introns and intergenic sites, and very few to promoter sequences (Figure 3E). Interestingly, mean expression in 2–4 hour wild-type embryos for genes associated with *gd^7^* Up regions are higher than for genes associated with regions where CBP binding decreases or is lost in *gd^7^* (Figure 3F). Furthermore, there is a decrease in mean expression of genes associated with *gd^7^* Up regions during the course of development, whereas expression of genes where CBP binding is reduced or lost increases during development (Figure 3F). Taken together, these analyses suggest that the genomic regions where CBP-binding increases in *gd^7^* embryos are HOT intronic and intergenic enhancer sequences of highly expressed genes regulated by Medea and Dicheate.

Regions where CBP binding does not change between wild-type and *gd^7^* overlap best with Dorsal, GAF, and Medea, but some overlap is also found with other factors, in agreement with the high HOTness of these regions. Unchanged regions overlap well with most histone modifications, including H3K4me1 (enhancers) and H3K4me3 (active promoters), histone acetylation, but also with H3K27me3 (Polycomb-repressed chromatin) (Figure 3D). Unchanged peaks are found in introns and intergenic regions, but they are also common in promoters (Figure 3E). Like *gd^7^* Up regions, mean expression for genes associated with unchanged regions is high in 2–4 hour wild-type embryos, and decreases at later stages of development.

Regions where CBP binding is decreased in *gd^7^* embryos overlap mainly Dorsal and GAF (Figure 3C). Especially the *gd^7^* Down CBP peaks that are strongest in wild-type overlap Dorsal extensively, but show little overlap with other factors. Thus, in embryos where Dorsal fails to enter the nucleus (*gd^7^*), CBP binding is selectively reduced at regions where Dorsal, but few other factors bind. Interestingly, strong CBP-peaks that are decreased in *gd^7^* embryos (*gd^7^* Down) overlap more with the inactive chromatin mark H3K27me3 than with the active histone marks H3K18ac, H3K27ac, and H3K4me3 (Figure 3D). The regions where CBP binding decreases in *gd^7^* embryos are more often than other CBP regions found in promoters, but they also occur frequently in intronic and intergenic sequences (Figure 3E). Regions where CBP is reduced in *gd^7^* embryos are associated with genes that are medium-expressed in wild-type 2–4 hour embryos, but whose expression increase during development. Together, this indicates that regions where CBP-binding decreases in *gd^7^* embryos are associated with Dorsal-regulated tissue-specific genes that are silenced by repressive chromatin in tissues where they are not expressed (compare with the Dorsal-target gene *twist* below).

Unexpectedly, we found that the CBP peaks that are lost in *gd^7^* overlap virtually none of the top 300 sites for the 40 transcription factors (Figure 3C and Table S2), although 76% of these peaks overlap a Dorsal-binding region (Figure 1C). Thus, CBP binding is lost in *gd^7^* embryos from regions where Dorsal binds weakly in wild-type. Surprisingly, CBP-bound regions that are lost in *gd^7^* poorly overlap all types of histone modifications (Figure 3D). The *gd^7^* Lost regions are found in introns and intergenic sequences, but also in promoters (Figure 3E). Genes where CBP occupancy is lost in *gd^7^* show the lowest mean expression in wild-type 2–4 hour embryos, but increases at later stages of embryogenesis (Figure 3F). It appears that regions where CBP occupancy is lost in *gd^7^* embryos are found in low- and non-expressed genes that bind few other factors except Dorsal and GAF, and which are depleted in histone modifications.

Together, these analyses suggest that the four categories of CBP peaks occur at very different genomic sites. Regions where CBP occupancy is increased in *gd^7^* embryos are found in intronic and intergenic HOT regions of highly expressed Medea- and Dicheate-regulated genes, and are depleted of GAF. Unchanged regions are found in both promoters and enhancers of highly expressed genes, and are associated with many different factors. Regions with decreased CBP occupancy in *gd^7^* embryos are found in both promoters and enhancers of medium expressed genes. They are found predominantly where Dorsal, but few other factors bind and are regulated by H3K27 methylation. Finally, regions where CBP occupancy is lost are found in genes with low expression at regions devoid of chromatin modifications and most transcription factors except Dorsal and GAF.

### Differences in CBP binding between wild-type and mutant embryos correlate with changes in gene expression

**Figure 4:**
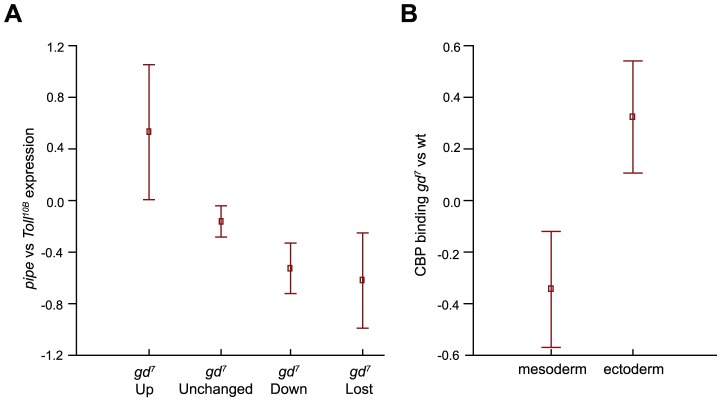
Differences in CBP occupancy between wild-type and *gd^7^* mutant embryos correlate with mean changes in gene expression. A) Differences in CBP occupancy between wild-type and *gd^7^* mutant embryos (no Dorsal in the nucleus) was compared to published gene expression changes in *pipe* mutant embryos (no Dorsal in the nucleus) versus *Toll^10B^* mutant embryos (high level of Dorsal in all nuclei) [25]. Squares indicate mean expression in *pipe* versus *Toll^10B^* mutant embryos, and whiskers correspond to 95% confidence intervals. There is a mean up-regulation of transcription at genes where CBP occupancy is increased at least 2-fold in *gd^7^* mutant embryos (*gd^7^* Up), and a mean decrease in expression where CBP occupancy is decreased or lost. The small decline in mean expression of unchanged regions is due to an overrepresentation of regions with CBP occupancy decreased less than 2-fold in the *gd^7^* Unchanged group. B) In *gd^7^* mutant embryos, a mean decrease in CBP occupancy at Dorsal mesoderm target genes (down-regulated in *pipe* and *gd^7^*), and a mean increase in CBP occupancy at Dorsal-target genes in the dorsal ectoderm (up-regulated in *pipe* and *gd^7^*) is observed. Mesoderm and ectoderm targets are defined in [25]. Squares indicate mean CBP binding in *gd^7^* versus wild-type embryos, and whiskers correspond to 95% confidence intervals.

Dorsal-regulated genes have previously been identified by comparing the difference in gene expression in *pipe* versus *Toll^10B^* mutant embryos [25]. In *pipe* mutants, the proteolytic cascade leading to Toll ligand activation is not initiated, and just as in *gd^7^* mutants, Dorsal protein does not enter the nuclei in these embryos. *Toll^10B^* mutants on the other hand contain high levels of Dorsal in all nuclei. The difference in gene expression between the two represents Dorsal-dependent expression, and was plotted against changes in CBP occupancy between wild-type and *gd^7^* mutant embryos (Figure 4A). All genes on the arrays used by Stathopoulos et al. [25] that overlapped a CBP bound region in wild-type or that had a CBP region within 500 bp were considered. This shows that at sites where CBP occupancy is increased in *gd^7^* mutants compared to wild-type, the corresponding genes are on average up-regulated in *pipe* mutant embryos. Genes associated with regions where CBP occupancy does not change in *gd^7^* embryos do not alter their expression significantly between *pipe* and *Toll^10B^* mutants. By contrast, at regions where CBP occupancy is reduced or lost in *gd^7^* mutants compared to wild-type, mean gene expression is decreased (Figure 4A). We then examined the difference in CBP occupancy between *gd^7^* mutants and wild-type at regions that overlap Dorsal binding at mesoderm-targets (down-regulated in *pipe* and *gd^7^*) and dorsal ectoderm-targets (up-regulated in *pipe* and *gd^7^*) as defined in [25]. Figure 4B shows that mean CBP occupancy is decreased at Dorsal-targets in the mesoderm and increased at targets in the dorsal ectoderm in *gd^7^* mutant embryos. Taken together, our results show that differences in CBP occupancy correlate well with changes in gene expression genome-wide.

### Binding of CBP to silent genes does not lead to histone acetylation

We wanted to compare changes in CBP occupancy to histone acetylation levels, and therefore looked closer at some of the best known Dorsal targets in the mesoderm and dorsal ectoderm. We compared histone modifications with CBP binding at these genes in *gd^7^*, *Toll^rm9/rm10^*, and *Toll^10B^* mutant embryos by ChIP-qPCR. In these mutant backgrounds the entire embryo is converted to dorsal ectoderm (*gd^7^*), neuroectoderm (*Toll^rm9/rm10^*), or mesoderm (*Toll^10B^*). We normalized the binding of Dorsal, CBP, and histone modifications to two intergenic regions, selected based on the absence of protein binding and histone modifications, and plotted the fold enrichment relative these intergenic sites. The histone modifications were additionally normalized to histone H3 levels (Table S6).

**Figure 5:**
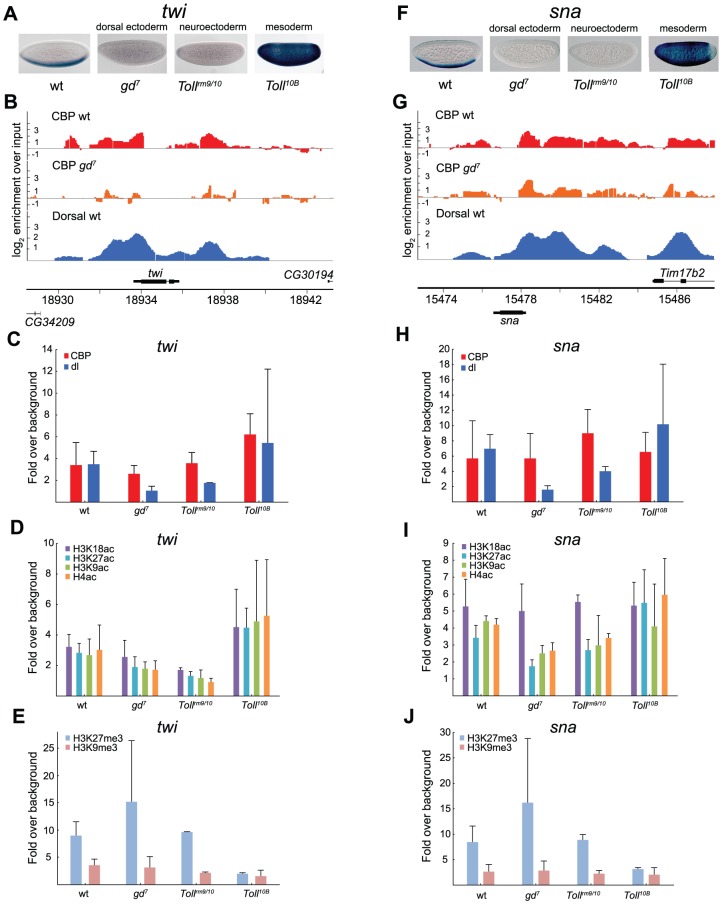
CBP occupancy at Dorsal-target genes in the mesoderm does not always correlate with changes in Dorsal occupancy or gene expression. A and F) *In situ* hybridization with digoxigenin-labeled *twist* (*twi*) and *snail* (*sna*) RNA probes in 2–4 hour old wild-type (wt) and mutant embryos that alter the Dorsal protein gradient, resulting in conversion of the entire embryo into dorsal ectoderm, neuroectoderm, or mesoderm. Embryos are oriented with anterior to the left and dorsal up. B and G) ChIP-seq peaks for CBP in wild-type (wt) and *gd^7^* mutant embryos (raw data without cut-off), as well as Dorsal ChIP-chip peaks in wild-type are shown for the *twi* and *sna* loci. Occupancy is plotted as log_2_-fold enrichment over input. C–E and H–J) ChIP-qPCR of CBP, Dorsal, H3K9ac, H3K18ac, H3K27ac, and H4ac, as well as H3K27me3 and H3K9me3 at the Dorsal target genes *twi* and *sna* in wild-type (wt) and mutant embryos. Occupancy is plotted as enrichment relative the average of two negative control loci (intergenic regions), and the histone acetylations normalized against histone H3 occupancy. ChIP was performed on 2–7 independent biological replicates for each genotype. Error bars indicate standard deviation, see Results for T-tests. At the *twi* locus, changes in CBP occupancy closely follow changes in Dorsal occupancy and gene expression, whereas at the *sna* locus, CBP occupancy is independent of Dorsal and gene expression.

In the mesoderm, Dorsal targets such as *twi* and *sna*, are activated by high levels of Dorsal. These genes are therefore not expressed in *gd^7^* and *Toll^rm9/rm10^* mutant embryos that contain no Dorsal or an intermediate concentration of nuclear Dorsal in the entire embryo. In *Toll^10B^* mutant embryos Dorsal is present in high amounts and *twi* and *sna* are therefore expressed throughout the embryo (Figure 5A and 5F). Less CBP and less histone acetylation on H3K9, H3K18, H3K27, and on histone H4 is found at the *twi* promoter in *gd^7^* embryos as compared to wild-type (Figure 5B–5D, T-test of all four histone acetylations in *gd^7^* vs wild-type, *p* = 0.0042). This is consistent with the genome-wide correlation of reduced CBP occupancy with lower gene expression and lack of Dorsal protein (Figure 3 and Figure 4). Interestingly, in *Toll^rm9/rm10^* embryos, there is a further reduction in histone acetylation without a corresponding decrease in CBP binding, as compared to *gd^7^* embryos (Figure 5D, T-test of histone acetylations in *Toll^rm9/rm10^* vs *gd^7^*, *p* = 0.020). This shows that the amount of CBP bound to a genomic region is not the only determinant of histone acetylation levels. In *Toll^10B^* embryos, *twi* expression is turned on in the entire embryo, and CBP as well as histone acetylations are present in high amounts (Figure 5B–5D, T-test of CBP in *gd^7^* vs *Toll^10B^*, *p* = 0.0028, histone acetylations in *gd^7^* vs *Toll^10B^*, *p* = 0.00072).

Unexpectedly, CBP binding to the *sna* promoter is not decreased in *gd^7^* mutant embryos compared to wild-type (Figure 5G and 5H), although Dorsal is absent and the gene not expressed (Figure 5F). By contrast, with the exception of H3K18ac, histone acetylation levels change in the different genetic backgrounds (Figure 5I, T-test of H3K9ac, H3K27ac, plus H4ac in wild-type vs *gd^7^*, *p* = 0.000011, *gd^7^* vs *Toll^rm9/rm10^*, *p* = 0.037, *gd^7^* vs *Toll^10B^*, *p* = 0.00033, *Toll^rm9/rm10^* vs *Toll^10B^*, *p* = 0.041). We therefore measured histone lysine methylation at Dorsal-target genes, a modification mutually exclusive to lysine acetylation. Interestingly, we observed high amounts of H3K27me3 in *gd^7^* and *Toll^rm9/rm10^* embryos (Figure 5E and 5J). This indicates that Polycomb-mediated repression is involved in keeping *twi* and *sna* off in the neuroectoderm and dorsal ectoderm. By contrast, H3K9me3, a mark for HP1-mediated repression, is absent on the *twi* and *sna* promoters in all three tissues (Figure 5E and 5J).

Interestingly, the high levels of H3K27me3 over the *sna* promoter in *gd^7^* embryos does not prevent CBP binding, indicating that Polycomb-repressed H3K27me3 chromatin is compatible with CBP binding. We therefore conclude that whereas CBP binding is not prevented by H3K27me3-repressed chromatin, histone acetylation is restricted. This conclusion is reinforced by our results from *Toll^rm9/rm10^* mutants, where *sna* is also repressed and H3K27me3 present, and CBP binding not significantly different from that in other genotypes. Relative *Toll^10B^* mutants, histone acetylation remains low in *Toll^rm9/rm10^* embryos (T-test of H3K9ac, H3K27ac, plus H4ac in *Toll^rm9/rm10^* vs *Toll^10B^*, *p* = 0.041), which is likely explained by the higher amounts of H3K27me3 in *Toll^rm9/rm10^* as compared to *Toll^10B^* embryos (Figure 5I and 5J, T-test of H3K27me3 in *Toll^rm9/rm10^* vs *Toll^10B^*, *p* = 0.0025). We note that H3K18ac levels in mutant embryos correlate with changes in CBP amount to a better extent than other histone acetylations, indicating that H3K18 may be a major *in vivo* target for CBP's HAT activity.

**Figure 6:**
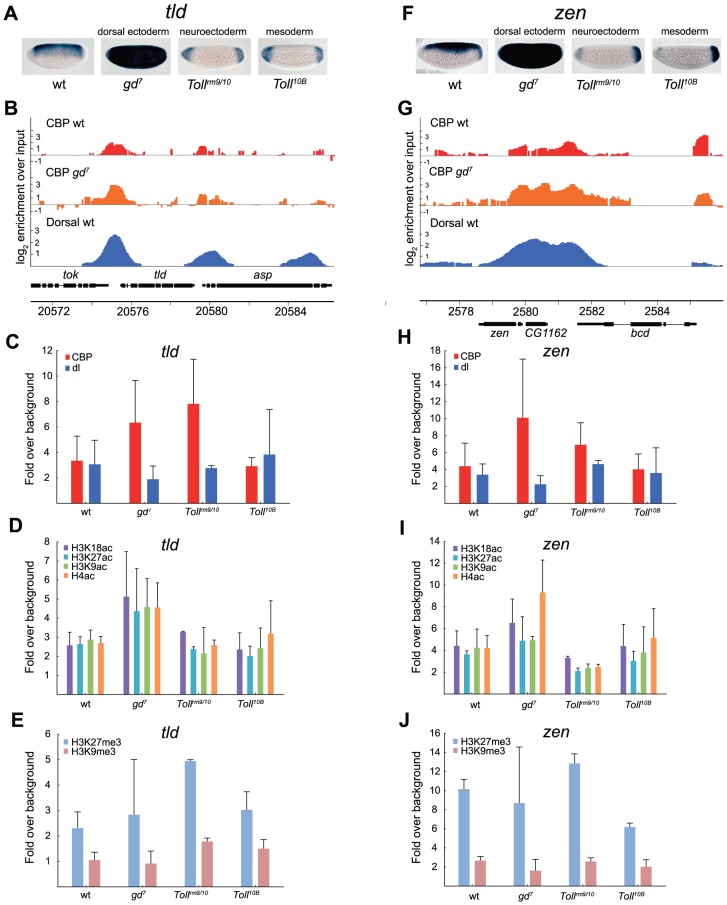
Histone acetylation by CBP at genes repressed by Dorsal is restricted by H3K27me3 in the neuroectoderm. A and F) *In situ* hybridization with digoxigenin-labeled *tolloid* (*tld*) and *zerknüllt* (*zen*) RNA probes in 2–4 hour old wild-type (wt) and mutant embryos that alter the Dorsal protein gradient. Embryos are oriented with anterior to the left and dorsal up. The Dorsal protein is converted to a repressor of *tld* and *zen* expression since the Dorsal-binding sites are flanked by AT-rich elements that bind co-factors and recruit the Groucho co-repressor. B and G) ChIP-seq peaks for CBP in wild-type (wt) and *gd^7^* mutant embryos (raw data without cut-off), as well as Dorsal ChIP-chip peaks in wild-type are shown for the *tld* and *zen* loci. Occupancy is plotted as log_2_-fold enrichment over input. C–E and H–J) ChIP-qPCR of CBP, Dorsal, H3K9ac, H3K18ac, H3K27ac, and H4ac, as well as H3K27me3 and H3K9me3 occupancy at *tld* and *zen* in wild-type (wt) and mutant embryos. Occupancy is plotted as in Figure 5. Error bars indicate standard deviation, see Results for T-tests. In *Toll^rm9/rm10^* mutant embryos, there is minimal histone acetylation at the *tld* and *zen* promoters although CBP binds to a significant extent. The high levels of H3K27 tri-methylation in these embryos may restrict histone acetylation by CBP and other HATs.

The dorsal ectoderm targets *tld* and *zen* are repressed by Dorsal, and therefore more highly expressed in *gd^7^* mutants that lack nuclear Dorsal, but completely repressed in *Toll^rm9/rm10^* and *Toll^10B^* mutants, except at the embryonic poles (Figure 6A and 6F). In *gd^7^* embryos, where these genes are expressed in more cells than in wild-type, binding of CBP is higher according to ChIP-seq (although not statistically significant by ChIP-qPCR) and acetylation of histones increases (Figure 6B–6D and 6G–6I, T-test of histone acetylations at *tld* in wild-type vs *gd^7^*, *p* = 0.00046, at *zen* in wild-type vs *gd^7^*, *p* = 0.0094). This is consistent with the genome-wide correlation of CBP occupancy and gene expression (Figure 4). In both *Toll^rm9/rm10^* and *Toll^10B^* mutant embryos, *tld* and *zen* are repressed. Surprisingly, although CBP binding is stronger in *Toll^rm9/rm10^* than in *Toll^10B^* embryos (T-test of CBP at *tld* in *Toll^rm9/rm10^* vs *Toll^10B^*, *p* = 0.037, but not significantly so at *zen*), there is equivalent amounts of histone acetylation in *Toll^10B^* embryos (Figure 6C, 6D and 6H, 6I). This may result from the high level of H3K27me3 in *Toll^rm9/rm10^* relative *Toll^10B^* embryos (Figure 6E and 6J, T-test of H3K27me3 at *tld* in *Toll^rm9/rm10^* vs *Toll^10B^*, *p* = 0.037, at *zen* in *Toll^rm9/rm10^* vs *Toll^10B^*, *p* = 0.0016). Taken together, our analysis of Dorsal-target genes in different tissues shows that CBP can bind to these genes when they are silenced, but that this does not result in high levels of histone acetylation.

In conclusion, our results suggest, 1) that CBP binding does not always correlate with gene expression or Dorsal binding, 2) that H3K18 acetylation levels closely follow CBP-binding, 3) that changes in histone acetylation can occur without a corresponding change in CBP binding, 4) that CBP binding is not prevented by the presence of H3K27me3 (Polycomb)-repressed chromatin, 5) but that H3K27me3-chromatin may restrict histone acetylation by CBP and other HATs.

## Discussion

CBP and the related p300 protein are widely used transcriptional co-activators in metazoans that interact with transcription factors of all major families [reviewed in 1], and they are for this reason believed to be present at many transcriptional regulatory regions. Therefore, CBP/p300 is expected to contribute to gene activation by most transcription factors, and not selectively regulate a subset of transcriptional programs. By contrast, we found that the gene regulatory networks controlling dorsal-ventral pattering of the *Drosophila* embryo are associated with CBP to a larger extent than e.g. anterior-posterior patterning.

### CBP is primarily involved in dorsal-ventral patterning of early *Drosophila* embryos

By comparison of CBP-bound regions in 2–4 hour old *Drosophila* embryos to previously mapped transcription factors [21], [22], we found an extensive overlap of CBP peaks with the key activator of dorsal-ventral patterning, the Rel-family transcription factor Dorsal. We then determined the genome-wide distribution of CBP in embryos where Dorsal cannot enter the nucleus (*gd^7^* mutants), and found that CBP peaks that overlap regions where Dorsal, but few other factors bind in wild-type are selectively reduced in *gd^7^* mutant embryos. Instead, strong CBP-bound regions in *gd^7^* mutants overlap best with regions bound by the Smad protein Medea, a mediator of Dpp-signaling. We and others have previously shown that signaling by the TGF-ß molecule Dpp is exceptionally sensitive to a small decline in the level of CBP in *Drosophila* embryos [10], [11]. Our present results are consistent with a function for CBP in the genomic response to Dpp-signaling.

**Figure 7:**
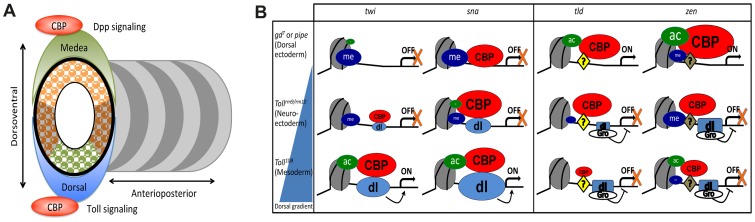
CBP may coordinate dorsal-ventral axis specification in *Drosophila* embryos. A) The *Drosophila* embryo is divided into segments along its anterior-posterior axis, whereas different germlayers arise along the dorsal-ventral axis. CBP occupancy genome-wide is over-represented at targets for the two key morphogens in dorsal-ventral patterning, Toll/Dorsal and Dpp/Medea. By contrast, anterior-posterior activators such as Bicoid and Caudal overlap the genomic distribution of CBP to a much smaller extent. B) Summary of histone modifications, Dorsal, and CBP occupancy at Dorsal-target genes in different tissues. An average of the four histone acetylations tested is colored green and H3K27 tri-methylation blue, CBP is colored red and Dorsal (dl) light blue. The size of the spheres represents protein/histone modification abundance and is drawn to scale. Dorsal represses *tld* and *zen* together with Groucho (Gro), whereas Zelda and possibly other factors (?) activate these genes. Although CBP binding is associated with gene expression and Dorsal binding genome-wide, CBP can also associate with silent genes. One reason that silent CBP-bound genes are not activated could be that H3K27me3-repressed chromatin restricts histone acetylation.

Less overlap of the CBP peaks is found with mapped activators of anterior-posterior patterning such as Stat92E, Fushi-tarazu (Ftz), Paired, Caudal, and Bicoid (Table S2). Previous work has indicated that CBP may function as a Bicoid co-activator. When Bicoid and CBP are expressed in S2 cells, they can interact, and Bicoid-mediated activation of reporter genes in these cells is influenced by CBP levels [26], [27]. We find that 43% of the 300 strongest Bicoid-binding regions overlap a CBP peak in wild-type embryos, indicating that CBP may participate in Bicoid-mediated activation *in vivo*. However, many of the Bicoid peaks are found in HOT regions that bind several transcription factors. Therefore, it may not be Bicoid that targets CBP to these sites. Furthermore, although the shape of the Bicoid gradient is slightly changed in embryos from the CBP hypomorph *nej^1^*, activation of Bicoid-target genes is not compromised by the decrease in CBP levels in *nej^1^* embryos [28]. Consistent with a non-essential function for CBP in Bicoid-mediated activation, there is no co-occupancy of CBP and Bicoid at the known target genes *hb*, *otd*, *kni*, and *eve* (Figure S3). Thus, although CBP may contribute to Bicoid-mediated activation of some target genes, it seems to make a more widespread contribution to Dorsal-mediated activation. In conclusion, both genetic and genomic evidence points to a particularly important function for CBP in controlling the two key events in dorsal-ventral patterning of *Drosophila* embryos, the Dorsal gene regulatory network and Dpp-signaling. Perhaps CBP serves to coordinate the Dorsal and Dpp pathways in dorsal-ventral patterning (Figure 7A).

### CBP binds strongly to enhancers of upregulated genes in mutant embryos

In embryos where Dorsal cannot enter the nucleus, we found places where CBP occupancy is increased, unchanged, decreased or lost. Regions that are unchanged bind several transcription factors, evident in their high HOTness, indicating that in the absence of Dorsal, other factors maintain CBP binding at these sites (Figure 3). Surprisingly, regions where CBP binding is increased are even HOTer, and therefore associated with even more factors in wild-type embryos. Although many CBP peaks in the genome are found where also GAF binds, the regions where CBP occupancy increases in *gd^7^* embryos are lacking strong GAF binding, despite their high HOTness (Figure 3C). Perhaps binding of GAF to these sites is not compatible with proper regulation of the corresponding genes. Instead, many of these regions bind Medea and Dichaete, especially the places where CBP binding is strong already in wild-type. We show that in *gd^7^* embryos, Dpp/Medea-regulated genes are expressed in more cells, resulting in increased CBP signal (Figure 2). Our data indicate that also Dichaete-regulated genes are more highly expressed in *gd^7^* mutants, and that CBP-binding therefore increases at these regions.

Unexpectedly, median gene expression level of genes associated with *gd^7^* Up regions is high in wild-type embryos. Most genes associated with these regions increase in expression even further in the absence of Dorsal (Figure 4), in most cases probably due to an expansion in the number of cells expressing the gene. We therefore expected that these CBP-binding sites would be situated in promoter regions, and the increase in CBP binding a consequence of increased gene activity. However, we found that these sites are mainly found in intronic and intergenic regions associated with H3K4me1, a mark of transcriptional enhancers. This indicates that CBP becomes recruited to these enhancers to mediate gene activation, rather than passively associating with active gene regions.

### CBP occupancy in mutant embryos is related to HOTness and dependent on Dorsal

CBP occupancy in *gd^7^* embryos is reduced at regions where only few factors bind. The bigger the reduction in CBP occupancy compared to wild-type, the fewer the factors that are associated with such a region in wild-type, i.e. the lower the HOTness of the region (Figure 3B). CBP peaks that are reduced in *gd^7^* embryos are much more common at regions where Dorsal binds in wild-type compared to other factors, consistent with a requirement for Dorsal in targeting CBP to chromatin. Although not all of the *gd^7^* Down CBP peaks overlap the top 300 Dorsal-binding regions, 92% overlap Dorsal when all Dorsal-binding regions are considered (Table S5). Peaks where CBP is reduced in *gd^7^* embryos are found in several known Dorsal target genes, such as *twi*, *brk*, *htl*, and *Mef2* (Figure 5 and Figure S4). Furthermore, 10 of the 20 strongest Dorsal peaks overlap a region where CBP binding is reduced in *gd^7^* embryos. Together, these data show that in early embryos, chromatin binding of CBP to many sites in the genome is dependent on Dorsal.

We found a number of genomic regions where CBP occupancy in *gd^7^* embryos is reduced to a level approaching background, the *gd^7^* Lost regions. These regions are mostly devoid of histone modifications and occupied by very few or none of the 40 transcription factors (Figure 3). The factors found at these regions bind at very low levels, indicating that they may not contribute to regulation of the corresponding genes at this stage of development. Further, most genes associated with the *gd^7^* Lost regions are expressed at very low levels or completely silent. These CBP-binding regions may therefore represent regulatory sequences that are poised for subsequent activation. Consistent with this interpretation, mean expression of the corresponding genes increases at later stages of development (Figure 3F). Why is CBP occupancy lost from these regions in *gd^7^* embryos? Perhaps these genes are not, and will not be expressed in the dorsal ectoderm, and are therefore not associated with CBP in *gd^7^* mutants that convert the entire embryo into dorsal ectoderm. Alternatively, CBP binding to these regions is dependent on Dorsal. Although binding is weak, Dorsal occupies many of these regions in wild-type (Figure 1C). It is possible that even small amounts of Dorsal is sufficient and necessary for CBP recruitment to these sites, and that CBP binding is consequently lost in the absence of Dorsal.

Although CBP occupancy is reduced predominantly at Dorsal-binding regions in *gd^7^* mutant embryos, expression of Dorsal target genes is also altered. The decrease in CBP occupancy in mutant embryos may therefore be a consequence of transcriptional inactivity, rather than a lack of recruitment by Dorsal. Indeed, as shown in Figure 4, CBP occupancy is on average reduced at down-regulated genes and increased at up-regulated genes. Therefore, although Dorsal and CBP occupancy often coincide, Dorsal may not directly recruit CBP to regulatory DNA sequences. However, there are also places where CBP occupancy is reduced without a corresponding change in gene expression. One such example is at the promoter of the *caudal* (*cad*) gene, which is co-occupied by Dorsal and CBP but where CBP binding is reduced more than two-fold in *gd^7^* embryos (Table S7), although the gene continues to be expressed [25]. Furthermore, as shown in Figure 1, Dorsal and CBP associate *in vivo*. We believe, therefore, that Dorsal may directly recruit CBP to many sites in the genome.

### Dorsal-independent CBP binding

As summarized in Figure 7B, there are also genomic sites where CBP occupancy is not dependent on either Dorsal or gene expression. Several known Dorsal target genes, including *sna*, *neur*, *ind* and *ths*, continue to associate with CBP in *gd^7^* embryos (Figure 5 and Figure S4). Although in general, HOTness is major determinant of CBP occupancy (Figure 3B), there is no big difference in HOTness of the Dorsal target gene regions where CBP-binding is reduced (e.g. *twi*, *htl*, *brk*) compared to Dorsal target gene regions where CBP binding is not changed (e.g. *sna*, *ind*, *ths*). What maintains CBP binding on these genes in the absence of Dorsal is not clear. Presumably, other factors recruit CBP to these sites in the absence of Dorsal, but we have not found a common factor for the regions where CBP binding is unchanged. We note, however, that GAGA-factor (GAF) associates with many of the CBP-binding regions in wild-type embryos, but much less with CBP-binding regions in *gd^7^* embryos. It is possible that GAF contributes to the recruitment of CBP to chromatin.

Dorsal is converted to a repressor when it binds in proximity to AT-rich sequences, and thereby prevents expression of dorsal ectoderm target genes in the neuroectoderm and mesoderm [17]–[19]. Consequently, these target genes, e.g. *dpp*, *zen*, and *tld*, are activated in all cells of *gd^7^* mutant embryos (Figure 2 and Figure 6). As expected, CBP occupancy increases at these target genes in *gd^7^* embryos, since more cells express the genes. The Zelda protein is a maternally contributed activator of these genes [29]–[31]. We have previously shown that in *nej^1^* embryos containing reduced amounts of CBP, *tld* expression is diminished, whereas *dpp* and *zen* expression remains unaffected [10]. It is possible, therefore, that more activators than Zelda contribute to activation of *tld*, *zen*, and *dpp* in the dorsal ectoderm. Until these factors are identified, it may not be possible to explain why *tld* expression is particularly sensitive to a reduction in CBP amount in early embryos.

### Silent genes bound by CBP are hypoacetylated

When Dorsal functions as a repressor, it recruits the Groucho co-repressor [19]. The yeast Tup1 protein, which is related to Groucho, was recently shown to block recruitment of co-activators to target genes [32]. By contrast, we find that CBP continues to associate with the *tld* and *zen* genes in the neuroectoderm although they are being repressed by Dorsal/Groucho (Figure 6). Groucho binds the histone deacetylase Rpd3 (HDAC1), which may be important for repression [33]. Indeed, we find that when *tld* and *zen* are repressed by Dorsal in the neuroectoderm and mesoderm, the genes are hypoacetylated despite the presence of CBP (Figure 6).

Contrary to the general trend, some genes recruit CBP even though they are silent. Why are these genes not activated? In the cases we have examined, histone acetylation is low despite the presence of CBP when the genes are not expressed (summarized in Figure 7B). Since lysine methylation and acetylation are mutually exclusive, we measured histone methylation at CBP-bound regions and found that Polycomb-repressed H3K27me3 chromatin is present at Dorsal-target genes in some tissues where these genes are not expressed. Although H3K27me3-decorated chromatin restricts DNA accessibility [34], we find that H3K27me3-chromatin does not preclude CBP binding, but restrains histone acetylation at these CBP-bound genomic sites. Interestingly, all histone acetylations that we measured are blocked by H3K27me3-chromatin, not only the mutually exclusive H3K27ac. This indicates that despite the ability of CBP to bind to genes enclosed in H3K27me3-chromatin, the histones are not accessible for acetylation by CBP and other HATs. Our data are consistent with a model for Polycomb silencing that allows access of proteins and pol II to DNA, but that restrains pol II elongation [reviewed in 35]. Perhaps high levels of histone acetylation are necessary for release of pol II from the promoter, for example by recruiting the bromodomain protein Brd4 that brings in the P-TEFb kinase to phosphorylate pol II [36].

### Regulation of CBP's HAT activity

In cells depleted of CBP and p300, global levels of H3K18ac and H3K27ac are greatly diminished whereas other histone acetylations remain unaffected, suggesting that these are *in vivo* targets of CBP acetylation [37], [38]. CBP can also acetylate H3K56, which occurs in response to DNA damage [39]. We find that H3K18ac and H3K27ac levels do not always correlate with changes in CBP occupancy at Dorsal target genes, although H3K18ac levels are most similar to CBP abundance (Figure 5 and Figure 6). In part, this can be explained by the presence of H3K27me3-chromatin, that precludes histone acetylation. However, in the neuroectoderm (*Toll^rm9/rm10^* embryos), the *twi* promoter contains less histone acetylation than in the dorsal ectoderm (*gd^7^* embryos) although H3K27me3 levels are reduced and CBP binding not decreased compared to dorsal ectoderm (Figure 5). Together, our results show that CBP's HAT activity is regulated by substrate availability, but that it may also be regulated by genomic context or signaling.

### Implications for enhancer prediction

Genome occupancy of CBP/p300 and H3K4me1 can be used to predict cis-regulatory DNA sequences [reviewed in 40]. However, what fraction of regulatory sequences that can be identified in this way is not known. We find that CBP binding to many known enhancer sequences that are active in early embryos is below our cut-off for high-confidence peaks, although we determined average CBP occupancy to be 1.73 times the genomic background at 97 previously described early embryonic enhancers [41]. Our results also show that CBP binding differs greatly between wild-type and mutant embryos, and that some gene regulatory networks rely on CBP to a much larger extent than others. Together, these results suggest that although CBP/p300 binding can be used to successfully identify transcriptional regulatory sequences, many enhancer sequences will be missed because they are not bound by CBP/p300 or bound at levels below criteria for high-confidence peaks. Even though mapping CBP/p300 binding in different cell-types will increase the number of putative regulatory sequences, we anticipate that a substantial number of enhancers will require alternative strategies for their identification, e.g. genome occupancy of other HATs [42].

In conclusion, we show that association of CBP with the genome is dependent on the number and types of transcription factors that bind the DNA sequence, that CBP preferentially associates with some gene regulatory networks, that CBP binding correlates with gene activity, but that CBP also binds silent genes without causing histone hyperacetylation.

## Materials and Methods

### Antibodies

A GST-dCBP amino acids (aa) 2540–3190 fusion protein was used to immunize rabbits and the resulting serum affinity-purified as described in [9]. This antibody has been used by modENCODE to map CBP binding during *Drosophila* development [5]. Its specificity was determined by Western blot in CBP RNAi-treated *Drosophila* S2 cells (Figure S1). The rabbit antibody was further compared to an affinity-purified guinea-pig anti-dCBP aa 1–178 serum [9]. ChIP experiments show that the two CBP antibodies precipitate DNA in a quantitatively similar manner (Figure S1). A guinea-pig anti-Dorsal serum (Su3) was provided by Christos Samakovlis (Stockholm University). The following antibodies recognizing histone modifications were used: H3 (ab1791), H3K9ac (ab4441), H3K9me3 (ab8898), H3K18ac (ab1191), H3K27ac (ab4729), H3K27me3 (ab6002), and IgG (ab6722) were from Abcam, and H4Ac (Upstate 06-598) was from Millipore.

### *Drosophila* embryo collections

Two- to four-hour old *Drosophila* embryos were collected on grape-juice plates, dechorionated, and used to prepare chromatin extracts as described below or fixed for *in situ* hybridization. Wild-type embryos were collected from *w^1118^* flies, and embryos where Dorsal fails to enter nuclei were collected from *gd^7^* homozygous mothers. Embryos with uniformly high levels of Dorsal in all nuclei were collected from *Toll^10B^* heterozygous females obtained directly from the balanced stock (*Toll^10B^*/TM3 Sb Ser/OR60). *Toll^rm9^/Toll^rm10^* trans-heterozygous females were used to collect embryos with intermediate levels of Dorsal in all nuclei.

### *In situ* hybridization

Whole-mount RNA in situ hybridization using digoxigenin-labeled probes was performed as described previously [43], [44].

### Co-immunoprecipitation

Two to four hour old embryos were dechorionated and crushed in a Dounce homogeniser in Lysis buffer (10 mM Tris pH 8, 140 mM NaCl, 1.5 mM MgCl_2_, 1% NP40, and proteinase inhibitors (Roche)). Lysate was shaken at 4°C and centrifuged. Pre-clearing and immunoprecipitation with the CBP antibody was done as previously described [45], except that Protein A Dynabeads (Invitrogen) were used. The immunoprecipitate was separated on 12% SDS-PAGE, transferred to nitrocellulose membrane, and probed with the Dorsal antibody diluted 1∶1000.

### ChIP

Two to four hour embryos were dechorionated, crushed in a Dounce homogeniser, and cross-linked with 1.8% formaldehyde in Buffer A1 (60 mM KCl, 15 mM NaCl, 15 mM Hepes pH 7.9, 4 mM MgCl_2_, 0.5 M DTT, 0.5% Triton X100, supplemented with proteinase inhibitor tablets, Roche) for 15 minutes at room temperature. The reaction was stopped with 0.225 M glycine and nuclei washed 3 times in Buffer A1 and once in Lysis buffer (140 mM NaCl, 15 mM Hepes pH 7.9, 1 mM EDTA, 0.5 mM EGTA, 0.1% sodium deoxycholate, 1% Triton X100, 0.5 M DTT, supplemented with proteinase inhibitor tablets, Roche). Nuclei were resuspended in Lysis buffer with 0.1% SDS and 0.5% N-lauroylsarcosine and sonicated in a Bioruptor (Diagenode). Chromatin extract was centrifuged to remove debris and diluted in an equal amount of lysis buffer, followed by snap freezing in liquid nitrogen and stored at −80°C.

A mix of Protein A and G Dynabeads (Invitrogen) blocked with 1 mg/ml BSA (Sigma Aldrich) were mixed with indicated antibodies. A bead-antibody complex was formed at 4°C for at least 4 hours. Beads with bound antibody were captured on magnet, and beads were resuspended in chromatin extract corresponding to 30–40 µl of embryos followed by incubation at 4°C over night. Beads were washed 5 minutes each with sonication buffer (50 mM Hepes, 140 mM NaCl, 1 mM EDTA, 1% Triton, 0.1% sodium deoxycholate, 0.1% SDS), WashA (as sonication buffer, but with 500 mM NaCl), WashB (20 mM Tris pH 8, 1 mM EDTA, 250 mM LiCl, 0.5% NP-40, 0.5% sodium deoxycholate) and TE. Beads were transferred in TE to new tubes and resuspended in Elution buffer (50 mM Tris pH 8, 50 mM NaCl, 2 mM EDTA, 0.75% SDS, 20 µg/ml RNase A, 20 µg/ml glycogen). Cross-linking was reversed at 68°C for at least 4 hours and Proteinase K treated followed by DNA purification with phenol-chloroform extraction and ethanol precipitation. The DNA was resuspended in 200 µl 0.1×TE.

The ChIP material was analysed either by qPCR or sent to the Uppsala Genome Center for SOLiD (TM) ChIP-Seq Library preparation (Rev date 18 March 2010), size selection (100–150 bp+adapters 90 bp ≈250 bp), and sequencing using SOLiD4 50 bp fragment run. Approximately 10 ChIPs for each genotype were pooled and used for the ChIP-seq libraries.

For ChIP-qPCR, duplicates with 2 µl DNA each were used for analysis by qPCR, using 300 nM primers (Table S8), and iQ SYBR green supermix, run on a CFX96 Real-Time system from BioRad. Average of the two duplicates were compared to input, and then normalized to the *Mi-2* locus or to two intergenic sites with background levels of binding. For histone antibodies, values were further normalized to total amount of histone H3.

### Processing of ChIP–seq data

Reads were aligned against the *Drosophila melanogaster* reference sequence (release 5) using the classical mapping in Applied Biosystems Bioscope software v1.2.1. The number of uniquely mapping reads was 21,667,438 (wild-type input), 17,131,635 (wild type CBP), 25,683,953 (*gd^7^* input) and 18,325,130 (*gd^7^* CBP). Average read-count per nucleotide was calculated for IP and input samples. For regions where the IP sample had at least the average read-count, a ratio of IP-input (in log_2_ scale) was calculated. If the read count in the input was below the average read-count (in the input sample) it was set to the average. All ratio values were then adjusted by reducing each value with the average read-count in IP minus the average read-count in input. This linear adjustment was to normalize for differences in sequencing depth of IP and input. Then the ratio value at an interval of 35 bp was extracted across the genome and median smoothed using a window size of 350 bp. Windows with fewer than 5 data points were discarded. The dynamic range of CBP in wild type was −2.0 to +5.1 and CBP in gd7 −1.8 to +5.1.

To calculate peaks and bound regions, the 5% highest ratio values in both wild-type and *gd^7^* were extracted, corresponding to a cut-off of 1.9 in wild type and 1.9 in *gd^7^*. Bound regions were then defined as regions of at least 200 bp and a region was extended as long as there was a value within 200 bp of the previous value. The value of each detected region was set to the average of the highest five consecutive ratio values. The center of the peak was set to the middle position of the five highest consecutive ratio values. When comparing CBP in *gd^7^* to CBP in wild type we did not normalize the two data sets to each other since the dynamic range and the 5% cut-off was more or less identical.

### Calculating overlap between data sets

When the overlap of bound regions of dataset X was to be compared to bound regions of other datasets first all regions of dataset X was used. Overlaps of at least one nucleotide were scored. Then an increasing cut-off for the binding values of data set X was applied. We used 20 cut-offs from the lowest to the highest binding value of dataset X in steps of maximum value minus minimum value then divided by 20. We plotted percent overlap using an increasing cut-off until about five percent of the binding regions of dataset X remained. In plots where only three cut-offs are shown, the cut-offs are; 1) no cut-off (all regions included), 2) an average of cut-offs 1 and 3, 3) the highest cut-off where only the ∼5% most enriched regions remained.

### Defining regions that have an altered CBP enrichment in *gd^7^* embryos

CBP peak values in *gd^7^* embryos were calculated (as described above) within each CBP bound region from WT embryos. In regions for which the *gd^7^* peak values were below 0.5 (data in log_2_ scale) CBP was considered lost. Next, a ratio of *gd^7^* and WT peak values was calculated. Regions with less than a two-fold difference (−1 to 1 in log_2_ ratio) were considered unchanged. Regions with more than two-fold higher *gd^7^* peaks were defined as going up and regions with more than two-fold higher WT peaks where defined as going down. When CBP peak values in WT embryos were determined within CBP bound regions in *gd^7^* embryos we found only two regions where no CBP could be detected in WT. We therefore did not define a class of regions unique for *gd^7^* embryos.

### Statistics

All statistics was performed using Statistica 10.0 (Statsoft). All reported T-tests are two-tailed without assuming equal variance. When multiple histone acetylations were compared between genotypes, all replicates from each acetylation were treated as one sample.

### Accession number

The ChIP-seq data is deposited in GEO under accession number GSE34221.

## Supporting Information

Figure S1 The affinity-purifed rabbit serum is CBP specific. A–B) Western blot and Coomassie-staining of CBP RNAi-treated *Drosophila* S2 cells. Primers with 5′T7 RNA polymerase-binding sites (Table S8) were used in PCR amplification of CBP and GFP cDNA. The 1002 bp (CBP) and 700 bp (GFP) PCR products were used to generate double stranded RNA for RNA interference using the Megascript RNAi kit (Ambion). 2×10^6^ S2 cells were washed twice in serum-free medium and resuspended in 750 µl serum-free medium before treatment with CBP dsRNA or GFP dsRNA. 37 mM of dsRNA was added and the cells were incubated for one hour at 25°C followed by adding 1.5 ml 15% FCS medium. After three days the cells were collected and washed in serum-free medium followed by a second dsRNA treatment, and harvested three days later. Cells were washed twice in PBS and resuspended in lysis buffer (50 mM Tris, pH 7.8, 150 mM NaCl, 1% Nonidet P-40, EDTA-free protease inhibitors) and lysed for 30 minutes at room temperature. Centrifugation at 13000 rpm for 5 min followed, and the supernatant was saved. The protein concentration was measured by the BCA protein assay kit (Thermo scientific). SDS-PAGE sample buffer was added, the samples denatured at 95°C for 5 min and centrifuged at 13000 rpm for 5 min before loading on 7.5% SDS-PAGE gels. The gel was either Coomassie-stained (B) or transferred to PVDF membrane (Bio Rad laboratories) at 30 V over night (A). The membrane was blocked in PBS containing 5% non-fat dry milk and incubated with the affinity-purified rabbit anti-dCBP serum (1∶200 in PBS containing 1% BSA) over night. The membrane was washed with PBS three times and incubated with HRP-coupled anti-rabbit antibody (1∶10000, DAKO) for one hour followed by ECL detection (GE Healthcare), and exposure to a Luminiscent Image Analyzer (LAS-1000plus, Fujifilm). Expression of the loading controls that we used to re-probe the membrane with was affected by the CBP RNAi treatment. We therefore compared total protein concentration between samples on Coomassie-stained gels. Arrow in A) points to full-length CBP, the other bands represent degradation products since they are also reduced in strength by CBP RNAi treatment. C) Comparison of ChIP signals obtained with the rabbit anti-dCBP aa 2540–3190 serum with ChIP signals from a guinea-pig anti-dCBP aa 1–178 serum [9]. Two to four hour old wild-type embryos were used for ChIP, and CBP targets with different levels of occupancy in ChIP-seq were analyzed by qPCR. Occupancy is plotted as enrichment relative the average of two negative control loci (intergenic regions). Mean fold enrichment and standard deviations from 3 independent biological replicates are shown.
(PDF)

Figure S2 CBP occupies Dpp-target genes in *gd^7^* mutant embryos. A) *In situ* hybridization of Dpp target genes *Race* and *pnr* in wild-type (*w^1118^*) and *gd^7^* derived embryos. Two to four hour old embryos were hybridized with digoxigenin-labeled probes and are oriented with anterior to the left, and dorsal up. Note the expanded expression of the Dpp target genes in *gd^7^* mutant embryos. B) ChIP-qPCR of CBP recruitment to Dpp target genes *Race* and *pnr*. Values are presented as fold over CBP binding at *Mi-2* whose expression and CBP binding is unaffected by the levels of Dorsal. As a negative control, the average of background CBP binding at two intergenic loci that do not bind CBP is included (IG). C–F) CBP and Medea occupancy overlap at the Dpp-target gene loci *Race* (*Ance*) (C), *pnr* (D), *GATAc* (*grn*) (E), and *tup* (F). CBP ChIP-seq peaks (as defined in Materials & Methods) in wild-type (wt) and *gd^7^* mutant embryos, as well as Medea ChIP-chip peaks in wt are shown. Occupancy is plotted as log_2_-fold enrichment over input.
(PDF)

Figure S3 CBP and Bicoid do not co-occupy Bicoid-target genes. A–D) CBP and Bicoid occupancy does not overlap at the Bicoid-target gene loci *eve* (A), *hb* (B), *kni* (C), and *otd (oc)* (D). CBP ChIP-seq peaks (as defined in Materials & Methods) in wild-type (wt) and *gd^7^* mutant embryos, as well as Bicoid ChIP-chip peaks in wt are shown. Occupancy is plotted as log_2_-fold enrichment over input.
(PDF)

Figure S4 CBP occupancy at Dorsal-target genes. In *gd^7^* embryos that lack nuclear Dorsal, CBP occupancy is reduced at some Dorsal-target genes (A–C), but relatively unaffected at other Dorsal-target genes (D–F) compared to wild-type. ChIP-seq peaks for CBP in wild-type (wt) and *gd^7^* mutant embryos (raw data without cut-off), as well as Dorsal ChIP-chip peaks in wild-type are shown for the *Mef2* (A), *htl* (B), *brk* (C), *neur* (D), *ind* (E), and *ths* (E) loci. Occupancy is plotted as log_2_-fold enrichment over input.
(PDF)

Table S1 Overlap between previously published CBP ChIP-seq peaks in 0–4 hour embryos [5] and regions bound by 40 sequence specific transcription factors [21], [22]. Overlap for all transcription factor binding sites or only the 300 most strongly occupied sites are shown in separate sheets.
(XLSX)

Table S2 Overlap between CBP ChIP-seq peaks from 2 to 4 hour old wt or *gd^7^* embryos and the 300 most strongly occupied regions for 40 sequence specific transcription factors [21], [22]. Overlap of ChIP-seq peaks with increasing cut-off is shown. Also, overlap of CBP peaks with the transcription factors after subdivision into four classes are shown; those with at least 2-fold higher CBP occupancy in *gd^7^* compared to wt (*gd^7^* Up), similar levels of CBP occupancy (*gd^7^* Unchanged), at least 2-fold less occupancy in *gd^7^* (*gd^7^* Down), and wt CBP peaks with no or background levels of binding in *gd^7^* (*gd^7^* Lost).
(XLSX)

Table S3 Overlap between CBP ChIP-seq raw data from 2 to 4 hour old wt or *gd^7^* embryos and all or the 300 most strongly occupied regions for 40 sequence specific transcription factors [21], [22]. Overlap of ChIP-seq raw data with increasing cut-off is shown.
(XLSX)

Table S4 Overlap between the 300 most strongly occupied regions for 40 sequence specific transcription factors [21], [22] and CBP ChIP-seq peaks from 2 to 4 hour old wt or *gd^7^* embryos.
(XLSX)

Table S5 Overlap between CBP ChIP-seq peaks from 2 to 4 hour old wt or *gd^7^* embryos and all regions for 40 sequence specific transcription factors [21], [22]. Overlap of ChIP-seq peaks with increasing cut-off is shown. Also, overlap of CBP peaks with the transcription factors after subdivision into four classes are shown; those with at least 2-fold higher CBP occupancy in *gd^7^* compared to wt (*gd^7^* Up), similar levels of CBP occupancy (*gd^7^* Unchanged), at least 2-fold less occupancy in *gd^7^* (*gd^7^* Down), and wt CBP peaks with no or background levels of binding in *gd^7^* (*gd^7^* Lost).
(XLSX)

Table S6 Binding of CBP, Dorsal, and indicated histone modifications at Dorsal target genes, expressed as fold over background. ChIP was performed on 2 to 4 hour old wt, *gd^7^*, *Toll^rm9/rm10^*, and *Toll^10B^* embryos. qPCR values were compared to input, and then normalized to two intergenic sites with background levels of binding. For histone antibodies, values were further normalized to total amount of histone H3. Values represent the average and standard deviation of the indicated number of independent ChIP experiments.
(XLSX)

Table S7 Peak position, peak value, and distance to closest TSS for CBP ChIP-seq peaks from 2 to 4 hour old wt or *gd^7^* embryos.
(XLSX)

Table S8 Primer sequences.
(PDF)
