# Supplementary material for: Preferential Genome Targeting of the CBP Co-Activator by Rel and Smad Proteins in Early Drosophila melanogaster Embryos
Source: PLoS Genet. 2012 Jun 21;8(6):e1002769. doi: 10.1371/journal.pgen.1002769 (PMC3380834; doi:10.1371/journal.pgen.1002769)
Supplement: Figure S1 — The affinity-purifed rabbit serum is CBP specific. A–B) Western blot and Coomassie-staining of CBP RNAi-treated Drosophila S2 cells. Primers with 5′T7 RNA polymerase-binding sites (Table S8) were used in PCR amplification of CBP and GFP cDNA. The 1002 bp (CBP) and 700 bp (GFP) PCR products were used to generate double stranded RNA for RNA interference using the Megascript RNAi kit (Ambion). 2×10^6^ S2 cells were washed twice in serum-free medium and resuspended in 750 µl serum-free medium before treatment with CBP dsRNA or GFP dsRNA. 37 mM of dsRNA was added and the cells were incubated for one hour at 25°C followed by adding 1.5 ml 15% FCS medium. After three days the cells were collected and washed in serum-free medium followed by a second dsRNA treatment, and harvested three days later. Cells were washed twice in PBS and resuspended in lysis buffer (50 mM Tris, pH 7.8, 150 mM NaCl, 1% Nonidet P-40, EDTA-free protease inhibitors) and lysed for 30 minutes at room temperature. Centrifugation at 13000 rpm for 5 min followed, and the supernatant was saved. The protein concentration was measured by the BCA protein assay kit (Thermo scientific). SDS-PAGE sample buffer was added, the samples denatured at 95°C for 5 min and centrifuged at 13000 rpm for 5 min before loading on 7.5% SDS-PAGE gels. The gel was either Coomassie-stained (B) or transferred to PVDF membrane (Bio Rad laboratories) at 30 V over night (A). The membrane was blocked in PBS containing 5% non-fat dry milk and incubated with the affinity-purified rabbit anti-dCBP serum (1∶200 in PBS containing 1% BSA) over night. The membrane was washed with PBS three times and incubated with HRP-coupled anti-rabbit antibody (1∶10000, DAKO) for one hour followed by ECL detection (GE Healthcare), and exposure to a Luminiscent Image Analyzer (LAS-1000plus, Fujifilm). Expression of the loading controls that we used to re-probe the membrane with was affected by the CBP RNAi treatment. We therefore compared total protein conce [file pgen.1002769.s001.pdf]

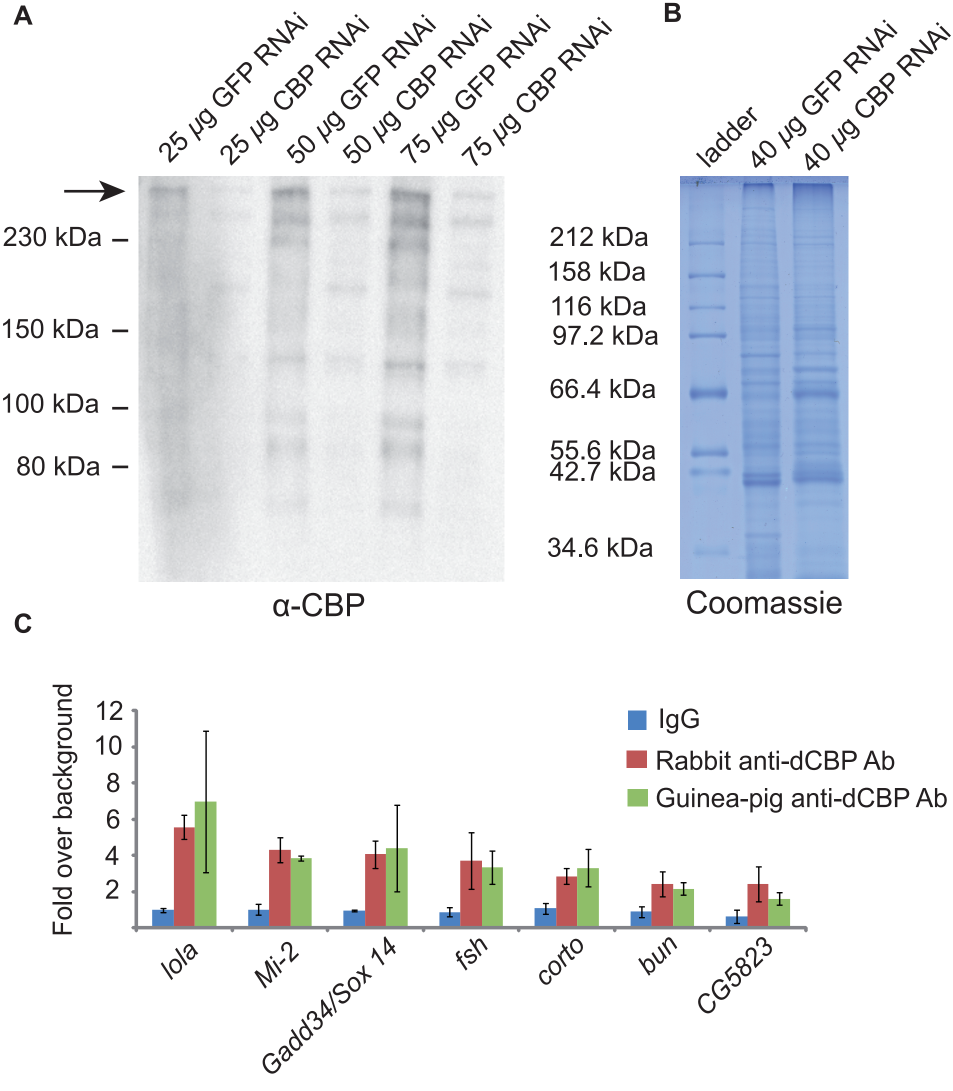
A
25 µg GFP RNAi
25 µg CBP RNAi
50 µg GFP RNAi
50 µg CBP RNAi
75 µg GFP RNAi
75 µg CBP RNAi
230 kDa
150 kDa
100 kDa
80 kDa
α-CBP
B
ladder
40 µg GFP RNAi
40 µg CBP RNAi
212 kDa
158 kDa
116 kDa
97.2 kDa
66.4 kDa
55.6 kDa
42.7 kDa
34.6 kDa
Coomassie
C
Fold over background
12
10
8
6
4
2
IgG
Rabbit anti-dCBP Ab
Guinea-pig anti-dCBP Ab
lola
Mi-2
Gadd34/Sox 14
fsh
corto
bun
CG5823
