# Supplementary figures and images for: Preferential Genome Targeting of the CBP Co-Activator by Rel and Smad Proteins in Early Drosophila melanogaster Embryos
Source: PLoS Genet. 2012 Jun 21;8(6):e1002769. doi: 10.1371/journal.pgen.1002769 (PMC3380834; doi:10.1371/journal.pgen.1002769)

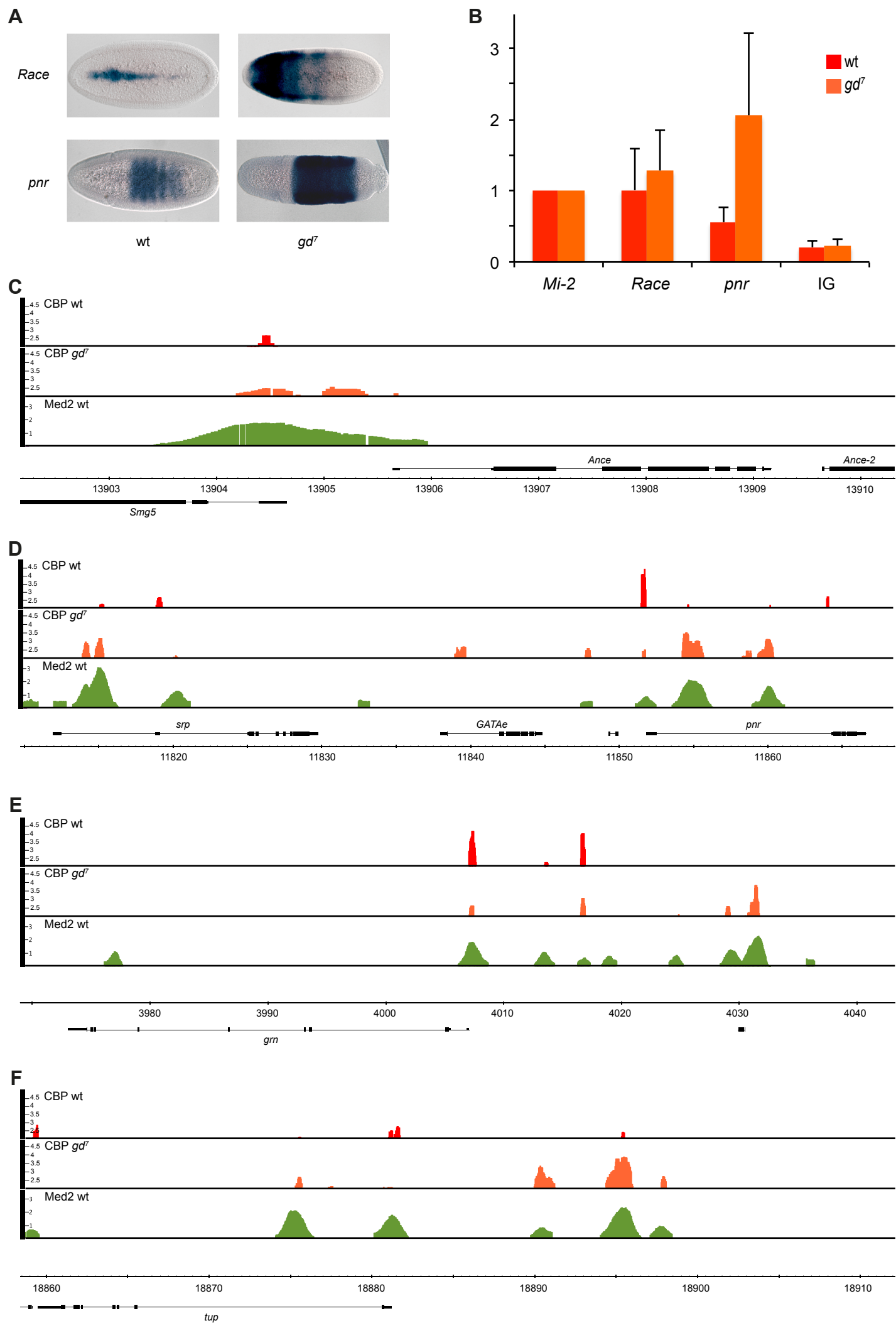

Supplement: Figure S2 — CBP occupies Dpp-target genes in gd7 mutant embryos. A) In situ hybridization of Dpp target genes Race and pnr in wild-type (w1118) and gd7 derived embryos. Two to four hour old embryos were hybridized with digoxigenin-labeled probes and are oriented with anterior to the left, and dorsal up. Note the expanded expression of the Dpp target genes in gd7 mutant embryos. B) ChIP-qPCR of CBP recruitment to Dpp target genes Race and pnr. Values are presented as fold over CBP binding at Mi-2 whose expression and CBP binding is unaffected by the levels of Dorsal. As a negative control, the average of background CBP binding at two intergenic loci that do not bind CBP is included (IG). C–F) CBP and Medea occupancy overlap at the Dpp-target gene loci Race (Ance) (C), pnr (D), GATAc (grn) (E), and tup (F). CBP ChIP-seq peaks (as defined in Materials & Methods) in wild-type (wt) and gd7 mutant embryos, as well as Medea ChIP-chip peaks in wt are shown. Occupancy is plotted as log2-fold enrichment over input. (PDF) [file pgen.1002769.s002.pdf]

A

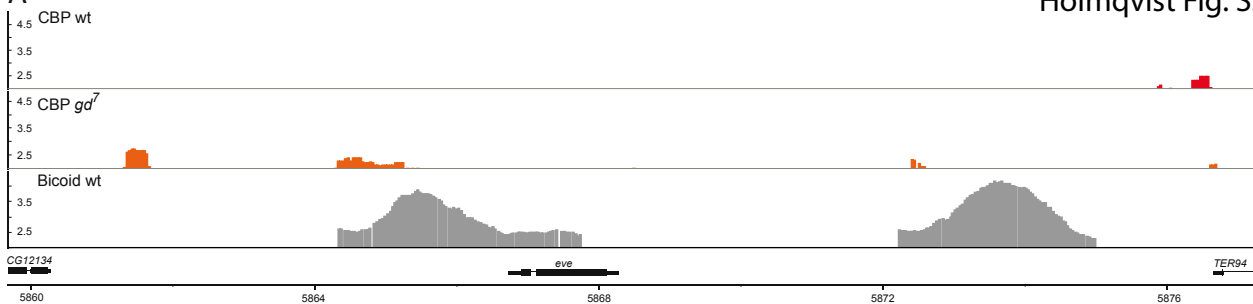

B

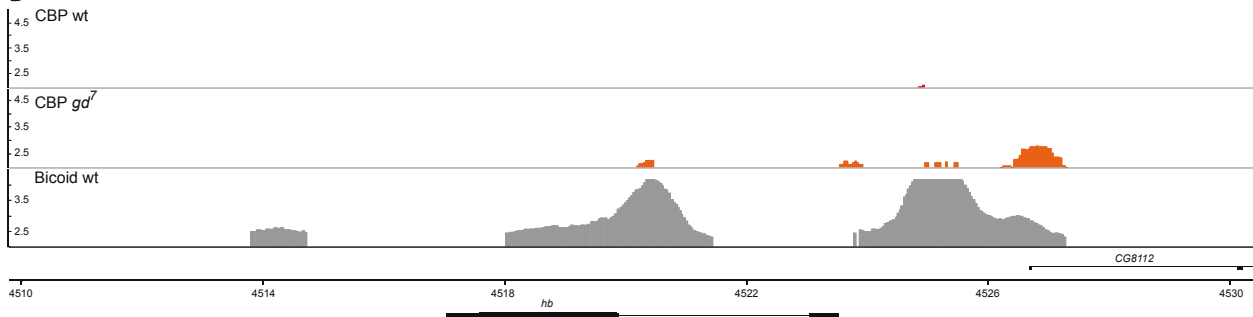

C

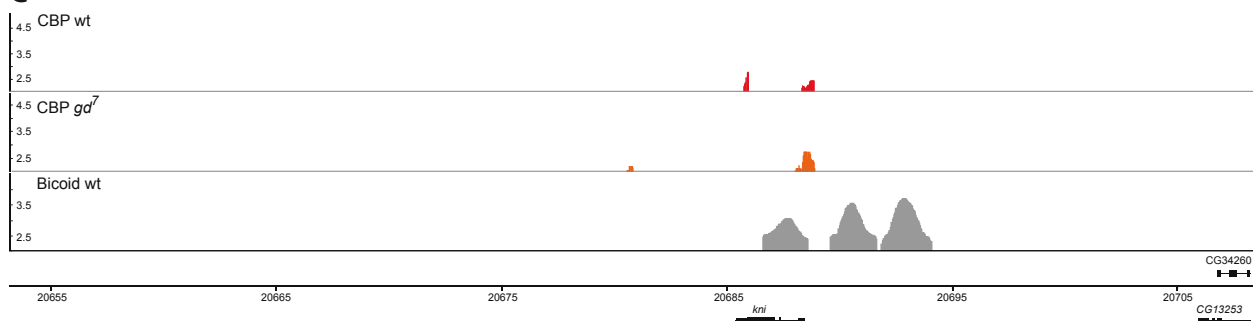

D

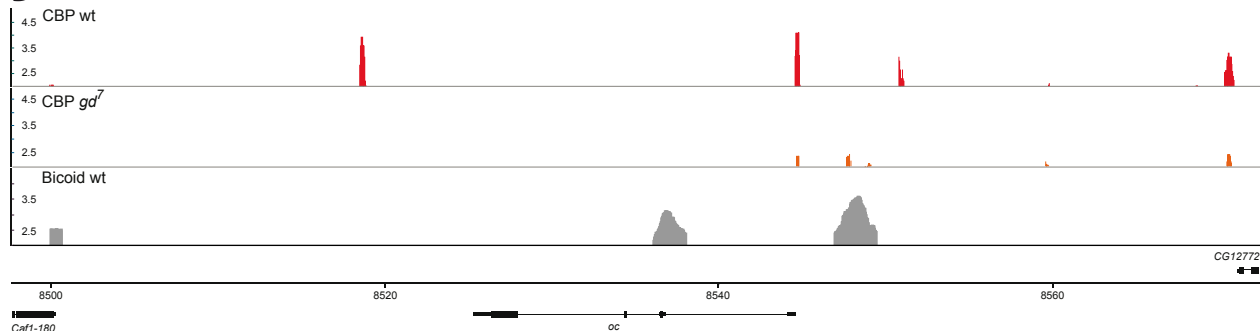

Supplement: Figure S3 — CBP and Bicoid do not co-occupy Bicoid-target genes. A–D) CBP and Bicoid occupancy does not overlap at the Bicoid-target gene loci eve (A), hb (B), kni (C), and otd (oc) (D). CBP ChIP-seq peaks (as defined in Materials & Methods) in wild-type (wt) and gd7 mutant embryos, as well as Bicoid ChIP-chip peaks in wt are shown. Occupancy is plotted as log2-fold enrichment over input. (PDF) [file pgen.1002769.s003.pdf]

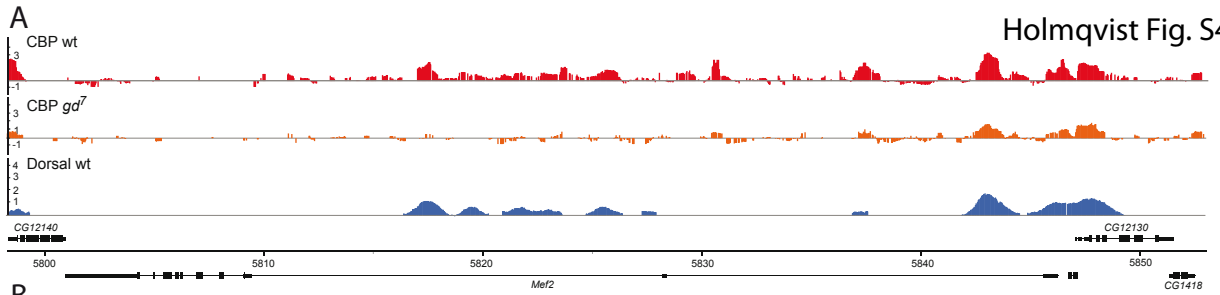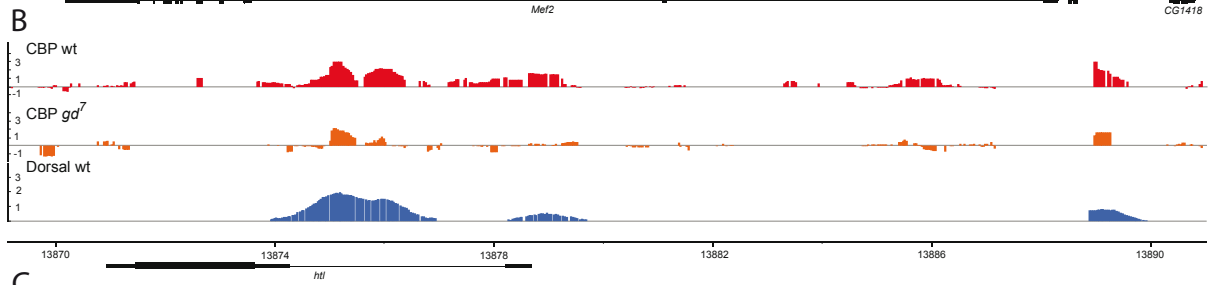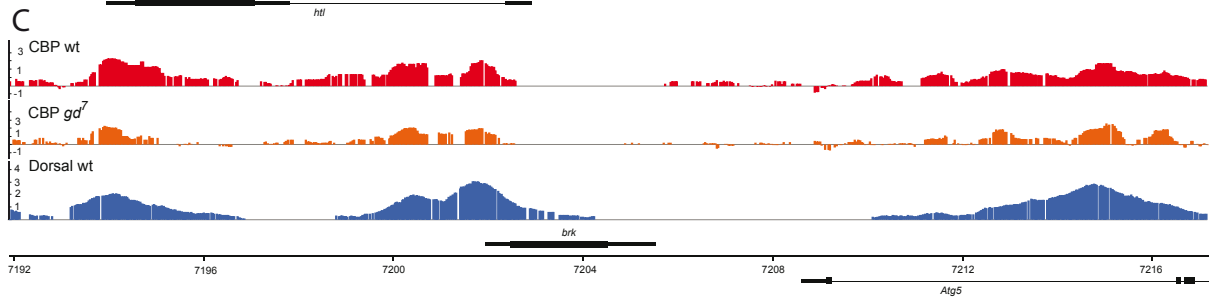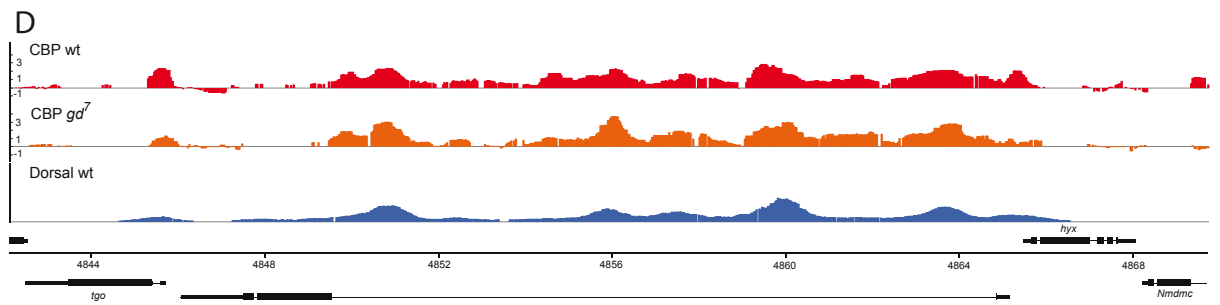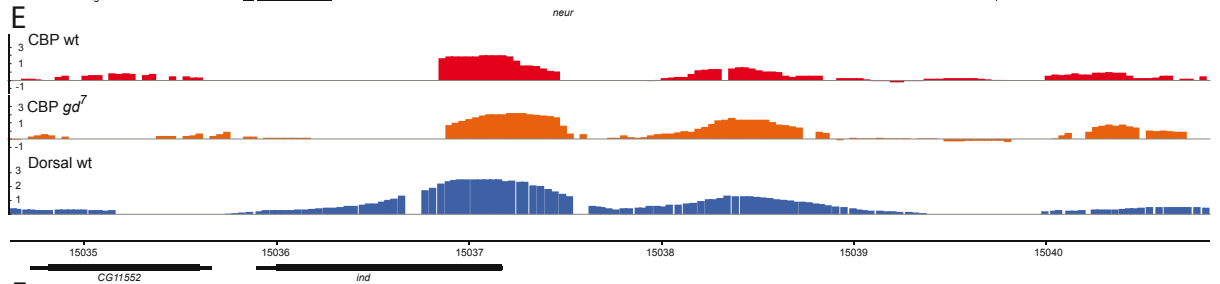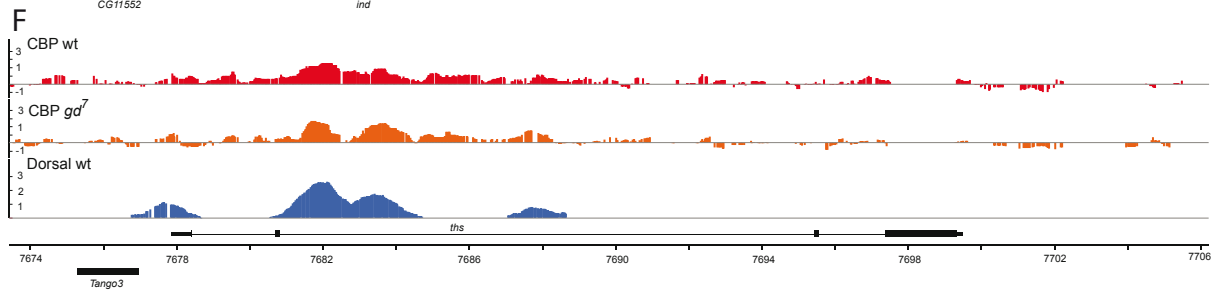

Supplement: Figure S4 — CBP occupancy at Dorsal-target genes. In gd7 embryos that lack nuclear Dorsal, CBP occupancy is reduced at some Dorsal-target genes (A–C), but relatively unaffected at other Dorsal-target genes (D–F) compared to wild-type. ChIP-seq peaks for CBP in wild-type (wt) and gd7 mutant embryos (raw data without cut-off), as well as Dorsal ChIP-chip peaks in wild-type are shown for the Mef2 (A), htl (B), brk (C), neur (D), ind (E), and ths (E) loci. Occupancy is plotted as log2-fold enrichment over input. (PDF) [file pgen.1002769.s004.pdf]
