# Supplementary material for: Preferential Genome Targeting of the CBP Co-Activator by Rel and Smad Proteins in Early Drosophila melanogaster Embryos
Source: PLoS Genet. 2012 Jun 21;8(6):e1002769. doi: 10.1371/journal.pgen.1002769 (PMC3380834; doi:10.1371/journal.pgen.1002769)
Supplement: Table S8 — Primer sequences. (PDF) [file pgen.1002769.s012.pdf]

Primer name	sequence
**ChIP-qPCR**	
IG1c_F	AGCGTTGTGCGAAGGTAAAC
IG1c_R	ATGGCCATTCCCCAAGGTCAT
IG2c_F	ATACACTTGGTAGAGGGCGTGCTAAAA
IG2c_R	CCCGCTGAAAAGGGGAAACAC
Mi-2(B)_F	CAGTGGAATGCTCAAGAGAAAAGACAA
Mi-2(B)_R	GCACCTAGGGGAATGGAATGGAT
Ush_F	AGTTCAGCGTCATCGTCATCGTTATC
Ush_R	GGCCTCCCTCGGTCTCTTTCA
Race_F	TTCACGTTTTCGCTCTTCCTTTGTT
Race_R	CAGCCATTGTTCCAGCCCTTCT
Pnr_F	ACAACTGGCAGGCGGATGATAAAT
Pnr_R	CGCAGAGCACGGCAGGACTT
Twi-CBP_F	CATTCGCCTGCGATTTTCTCG
Twi-CBP_R	CATCAGTTAGTTGCCAACCATCGTAAA
Sna PR F	AGCCGCATTTCCATTTCGTATTTC
Sna PR R	CGTGGCTTGTTTTGCTTGAGTTTC
Tld-CBP_F	AGCGCCATGGCTCTTGAATAGC
Tld-CBP_R	TTGCCCAGGATCAGGTGTTGC
zen-CBP_F	CGCGACATTTCCACACGGTTAT
zen-CBP_R	GGCCTTCCCATCCTCAGTCCT
fsh_F	TGATCCCCCGCAATTAGCACTC
fsh_R	GCGCGTTTGTCGAAAAATCGTAG
corto_F	GTCGGGGTGAGTGAACAATCCA
corto_R	ACCCCCGCGAGAAAAGAACA
Gadd34/Sox14_F	TAAAGGGGCACCGAATCAAATAAAG
Gadd34/Sox14_R	CGTGCCAGAGACCGAAAGAGC
lola_F	CGACGCAAATGTGACGGTGACTG
lola_R	CTCGCTCGCTCGCTCCATCC
bun_F	GGCGATGTGGCGTGTAGCAAGTT
bun_R	GTCCGGTGGTCAGATACAGATACAGAA
CG5823_F	CCGCTACAGTGGATGCCCCTAAT
CG5823_R	CGCACACGGTACCACTAAACTCTTCA
CG43143_F	GAGAGCGAAGGCAAACAAACGAAAAT
CG43143_R	TGCGGCCTCCAGACCCATACA
**CBP RNAi**	
nej-R1_F	ctaatacgactcactatagggagCAGCATTGTGCCTCTTCGC
nej-R1_R	ctaatacgactcactatagggagCATCAGGACACTGGAACCG
GFP_F	taatacgactcactatagggagaGGTGAGCAAGGGCGAGGAGCTGTTC
GFP_R	taatacgactcactatagggagaATGCCGAGAGTGATCCCGGCGGCGG
